# Porphyrin–azoheteroarenes: synthesis, photophysical, and computational studies

**DOI:** 10.1039/d5ra03790e

**Published:** 2025-08-08

**Authors:** Sahana Nagesh Shet, Vighneshwar Ganesh Bhat, Swathi S. G., Udaya Kumar Dalimba, Vijayendra S. Shetti

**Affiliations:** a Department of Chemistry, National Institute of Technology Karnataka Surathkal-575025 India shettivs@nitk.edu.in

## Abstract

Azobenzenes (Ph–N=N–Ph) are well-known photochromic compounds with widespread applications. Replacing one or both phenyl rings of azobenzenes with heteroarenes leads to a new class of compounds known as azoheteroarenes (Het–N=N–Ph/Het). Azoheteroarenes have gained attention as promising alternatives to traditional azobenzenes in the field of photopharmacology due to their ability to undergo photoswitching under visible light. Interestingly, the chemistry of porphyrin-containing azoheteroarenes has been underexplored. In this study, we present the synthesis of hitherto unknown hybrid molecules: porphyrin–azopyrroles (porphyrin–N=N–pyrrole) and porphyrin–azoindoles (porphyrin–N=N–indole), which also feature porphyrins with five-membered *meso*-substituents, such as 2-furyl and 2-thienyl groups. The porphyrin–azoheteroarenes with *meso*-tris(2-furyl/2-thienyl) substitutions demonstrate red-shifted absorption and emission bands, more significant Stokes shifts, and smaller optical bandgaps compared to hybrids containing only *meso*-aryl groups. Additionally, these porphyrin–azoheteroarenes exhibit higher fluorescence emission intensities than their corresponding precursor porphyrins.

## Abstract 2

This paper presents the synthesis, experimental studies, and computational analysis of a new class of hybrid molecules called porphyrin–azopyrroles and porphyrin–azoindoles, which feature porphyrins containing five-membered *meso*-substituents.

## Graphical abstract

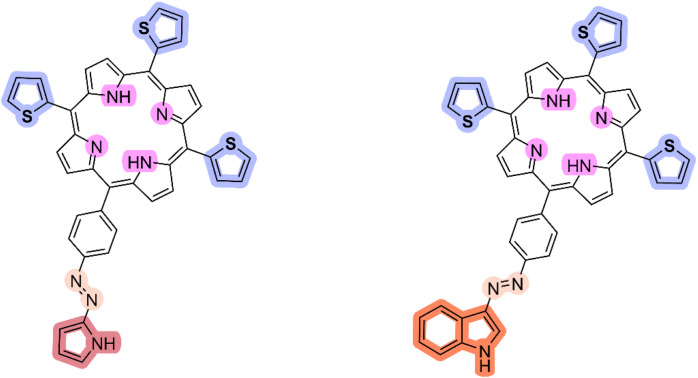

## Introduction

Azobenzenes (Ph–N=N–Ph) and their derivatives are a diverse group of photochromic compounds known for their ability to undergo light-induced reversible *E*/*Z* isomerization.^1–5^ These compounds are used as photoswitches in various fields, including medicine, optical storage, liquid crystals, and electronic devices.^6–8^ When one (Ph–N=N–Het) or both phenyl rings (Het–N=N–Het) of azobenzenes are replaced with heteroarene (heterocycle) moieties, a new class of molecules called azoheteroarenes is formed.^9–11^ Studies have indicated that azoheteroarenes containing pyrrole, indole, pyridine, pyrazole, imidazole, thiazole, and benzothiazole are credible alternatives to traditional azobenzene derivatives, as they exhibit photoswitching under visible light.^12–16^ The biological significance of heteroarenes,^17,18^ combined with their capability for visible-light photoswitching, makes azoheteroarenes promising candidates for photopharmacology applications. A recent perspective paper by Li and Zhang highlights the latest developments in this area.^19^

Porphyrins are often referred to as the ‘pigments of life’ due to their vital roles in various essential biological functions.^20,21^ Structurally, porphyrins are aromatic macrocycles that contain 18 π-electrons in their shortest conjugation pathways. Synthetic variants of porphyrin hybrids have been utilized in fields such as molecular electronics, nonlinear optical materials, catalysis, and biomedicine.^22–25^ Among these, porphyrin–azoarene hybrids (porphyrin–N=N–arene) have been sporadically investigated as model systems to study energy/electron transfer dynamics and as molecular spin switches.^26–31^ However, porphyrin–azoheteroarene hybrids (porphyrin–N=N–Het) have rarely been explored.^32,33^ Additionally, there are currently no examples of porphyrin–azoheteroarenes that feature porphyrins with five-membered *meso*-substituents, such as 2-furyl and 2-thienyl groups on their macrocyclic ring. Incorporating these five-membered *meso*-substituents onto the porphyrin ring enhances the macrocyclic π-delocalization, resulting in red-shifted absorption and emission bands.^30,34^

Building on these insights, we developed a synthetic strategy to create two new types of hybrid molecules: porphyrin–azopyrroles (porphyrin–N=N–pyrrole) and porphyrin–azoindoles (porphyrin–N=N–indole). These molecules fall under the broader category of porphyrin–azoheteroarenes. Our approach also includes porphyrins with three five-membered *meso*-substituents, specifically *meso*-tris(2-furyl/2-thienyl) substituted porphyrins, in addition to *meso*-tris(*p*-tolyl) substituted porphyrins, as we designed these hybrids. This paper presents the synthesis, photophysical, anion-binding, and computational studies of these hybrid molecules.

## Results and discussion

### Synthesis

**Scheme 1:**
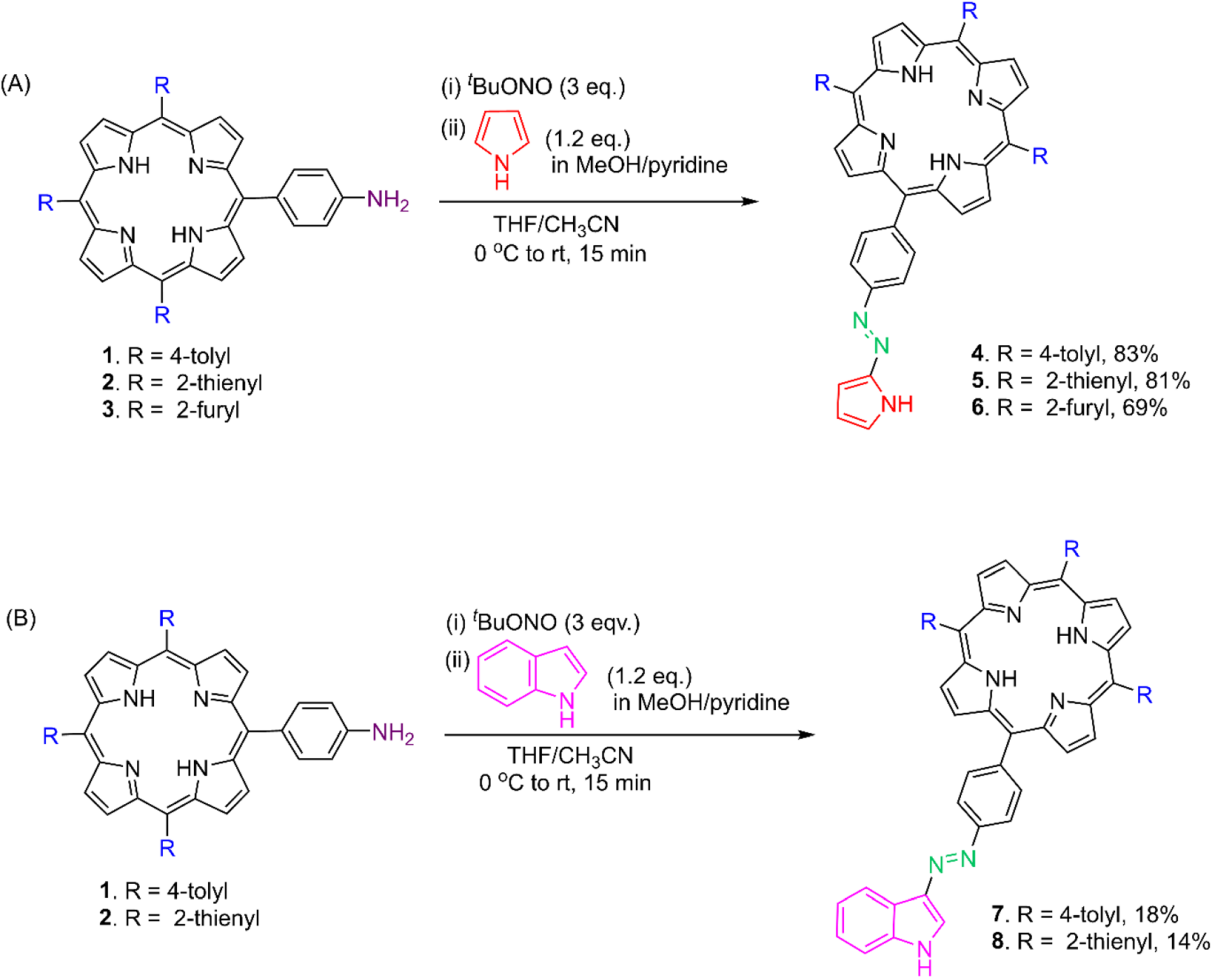
Synthesis of (A) porphyrin–azopyrroles 4–6 and (B) porphyrin–azoindoles 7 & 8.

The chemical syntheses of two classes of porphyrin–azoheteroarenes, namely, porphyrin–azopyrroles 4–6 and porphyrin–azoindoles 7 & 8, are delineated in Scheme 1(A and B).

**Fig. 1:**
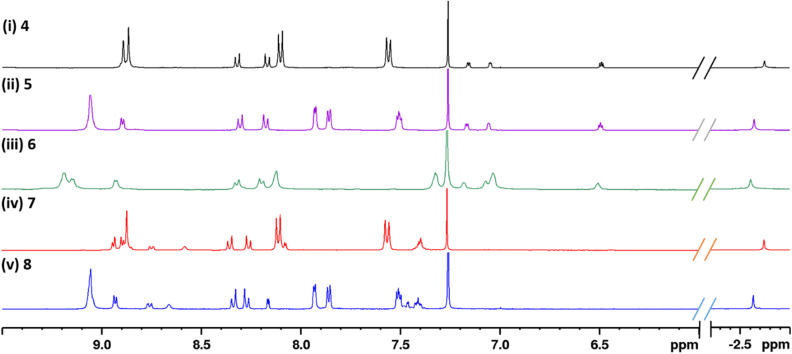
Partial ^1^H NMR spectra of hybrids (i) 4, (ii) 5, (iii) 6, (iv) 7, and (v) 8, recorded in CDCl_3_ at room temperature.

In a one-pot method, the *meso*-(4-aminophenyl) porphyrins 1–3 were first reacted with *tert*-butyl nitrite (^*t*^BuONO), followed by pyrrole/indole in the presence of a base to obtain the porphyrin–azoheteroarenes 4–8.^30,35^ The porphyrin–azopyrrole hybrids 4–6 were obtained in 69–83% yields after the column chromatographic purifications, whereas the isolated yield was lower for porphyrin–azoindoles 7 and 8. Our efforts to synthesize the *meso*-tris(2-furyl) substituted porphyrin–azoindole hybrid using this method were unsuccessful. All the hybrids 4–8 were characterized by NMR (^1^H & ^13^C) spectroscopy, and their molecular formulae assignments were based on HRMS data (Fig. S1–S15, SI). A stacked plot of the selected regions of ^1^H NMR spectra of hybrids 4–8 is presented in Fig. 1.

In the case of all the hybrids 4–8, the resonance signal corresponding to the porphyrin core NH was observed around *δ* −2.7 ppm as a singlet, and the signals due to the porphyrin core β-pyrrole protons appeared in *δ* 8.70–9.20 ppm region. The signals due to the *meso*-aryl ring connected to the N=N bridge in all the hybrids appeared as two downfield-shifted doublets around *δ* 8.20 and 8.35 ppm, compared to their corresponding precursor (4-aminophenyl) porphyrins (*δ* 7.07 and 7.99 ppm). In the case of 4 and 7, the signals due to the *meso*-tris(*p*-tolyl) groups attached to the porphyrin ring appeared as a singlet at *δ* 2.7 ppm and as two doublets at *δ* 7.56 and 8.10 ppm. Similarly, the signals due to the *meso*-tris(2-thienyl) groups in hybrids 5 and 8 and due to *meso*-tris(2-furyl) groups in hybrid 6 appeared as three multiplets in the region *δ* 7.49–7.93 and 7.03–8.11 ppm, respectively. The signals corresponding to the pyrrole moiety linked to the porphyrin ring *via* an azo-bridge appeared in three sets around *δ* 6.48, 7.04, and 7.16 ppm in 4–6. Among these, the signal at *δ* 6.48, 7.04 ppm are due to the β-H of pyrrole (*vs. δ* 7.25 ppm in free pyrrole) and, the signal at *δ* 7.16 is due to the free *α*-H of pyrrole (*vs. δ* 6.14 ppm in free pyrrole). In the case of indole hybrids 7 and 8, the signals due to the azo-linked indole moiety (*δ* 7.4–8.8 ppm) were downfield shifted compared to the free indole (*δ* 7.04–7.64 ppm). The NH signal of the indole appeared as broad singlet around *δ* 8.6 ppm in both hybrids.

### Photophysical properties

**Fig. 2:**
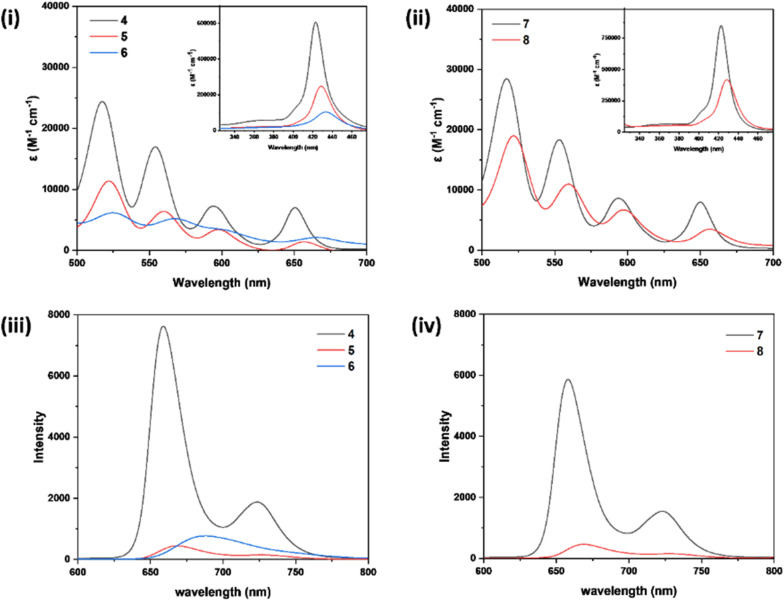
Comparison of (i) Q-band absorption spectra of hybrids 4 (black line), 5 (red line) and 6 (blue line), (ii) Q-band absorption spectra of hybrids 7 (black line) and 8 (red line) (Soret-bands are shown in inset), (iii) emission spectra of hybrids 4 (black line), 5 (red line) and 6 (blue line), (iv) emission spectra of hybrids 7 (black line) and 8 (red line). All the spectra except the Soret bands shown in (i) & (ii) were recorded in 1 × 10^−5^ M solution of toluene and, for the Soret-band measurement, the concentration used was 1 × 10^−6^ M.

The UV-vis absorption, steady-state/time-resolved emission studies of all the hybrids 4–8 and their respective precursor porphyrins 1–3 were performed in toluene solutions, and the data associated with these studies are presented in Tables 1 & 2. The absorption and emission profiles of all the hybrids are shown in in Fig. 2. An excitation wavelength, *i.e.*, *λ*_ex_ = 400 nm, was used while performing steady-state fluorescence studies. The singlet-state lifetime measurements were made using the Time Correlated Single Photon Counting (TCSPC) technique (*λ*_ex_ = 510 nm) while observing the emission maxima. The fluorescence decay curves (Fig. S16, SI) were fitted to single or biexponential curves to determine the lifetime data, and radiative (*k*_rad_) and non-radiative decay constants (*k*_nr_) were subsequently calculated.^36^

**Table 1:** Absorption data of porphyrin-azoheteroarene hybrids 4–8 and their corresponding precursors 1–3 recorded in toluene (the concentration used was 1 × 10^−6^ M and 1 × 10^−5^ M for Soret band and Q-bands measurements, respectively)

Compound	Soret band *λ* [nm] (log *ε*)	Q bands *λ* [nm] (log *ε*)
1	424 (5.73)	518 (4.25), 555 (4.05), 594 (3.67), 651 (3.65)
2	430 (5.65)	523 (4.24), 561 (4.01), 598 (3.75), 658 (3.42)
3	432 (5.03)	524 (3.75), 569 (3.72), 663 (3.11)
4	423 (5.78)	517 (4.38), 554 (4.23), 594 (3.86), 650 (3.84)
5	429 (5.39)	522 (4.05), 560 (3.81), 598 (3.53), 657 (3.14)
6	433 (5.01)	525 (3.79), 568 (3.71), 666 (3.33)
7	423 (5.92)	517 (4.45), 553 (4.26), 594 (3.93), 650 (3.90)
8	429 (5.62)	522 (4.27), 560 (4.04), 598 (3.82), 657 (3.54)

**Table 2:** Emission data of porphyrin-azoheteroarene hybrids 4–8 and their corresponding precursors 1–3 recorded in toluene (the concentration used was 1 × 10^−5^ M)

Compound	*λ*_em_ [nm]	*Φ*_f_	*τ*_f_ [ns]	*k*_rad_ (10^6^ s^−1^)	*k*_nr_ (10^6^ s^−1^)	Stokes shift *Q*_*x*_(0,0) − Q^*^_x_(0,0) [cm^−1^]	*E*_opt_ [eV]
1	660, 725	0.093	9.35	9.94	97.00	209	1.890
2	671, 729	0.012	1.61	7.45	613.6	294	1.870
3	685	0.093	7.42	12.8	125.3	484	1.845
4	659, 724	0.23	9.63	23.8	79.95	210	1.896
5	668, 728	0.028	1.60	17.5	607.5	251	1.873
6	688	0.084	5.76	14.5	159.02	480	1.839
7	658, 723	0.18	9.91	18.16	82.74	210	1.896
8	669, 729	0.022	1.51	14.56	674.68	251	1.873

Hybrids 4 and 7, which contain *meso*-tris-(*p*-tolyl) substituted porphyrins, exhibited a strong Soret band at 423 nm along with four Q-bands in 517–650 nm region. In contrast, the hybrids 5 and 8, featuring *meso*-tris-(2-thienyl) substituted porphyrins, showed a strong Soret band at 429 nm and four Q-bands in 522–657 nm region. Similarly, the hybrid 6 containing *meso*-tris(2-furyl) substituted porphyrin displayed a Soret band at 433 nm and Q-bands in the 525–666 nm region. The red-shift of absorption bands noted in hybrids 5, 6, and 8 compared to hybrids 4 and 7 can be attributed to the presence of *meso*-tris(2-thienyl/2-furyl) substituents on the porphyrin macrocycle. Furthermore, the molar absorptivity coefficient values (*ε*) of all the absorption bands of porphyrin-azoindole hybrids 7 and 8 were greater than those of their corresponding azopyrrole analogs 4 and 5.

Among the azopyrrole hybrids 4–6, the *meso*-tris(2-thienyl) substituted hybrid 5 (668, 728 nm) and *meso*-tris(2-furyl) substituted hybrid 6 (broad band centered at 688 nm) displayed red-shifted emission bands compared to *meso*-tris(*p*-tolyl) substituted hybrid 4 (659, 724 nm). In the case of azoindole hybrids, compound 8 exhibited red-shifted emission bands (669, 729 nm) compared to 7 (658, 723 nm). Compared to their precursor porphyrins, the porphyrin-azoheteroarene hybrids, in general, exhibited considerably enhanced fluorescence quantum yields (∼2–2.5 times). For example, the fluorescence quantum yield of hybrids 4 (0.23) and 7 (0.18) were significantly greater compared to their precursor porphyrin 1 (0.093). Similarly, the quantum yields of 5 (0.028) and 8 (0.022) were significantly higher than precursor porphyrin 2 (0.012). Among the pyrrole hybrids, a considerably more significant Stokes shift was observed for hybrid 6 (480 cm^−1^) compared to 4 and 5 (210 and 251 cm^−1^, respectively), and interestingly, this shift was even greater than its earlier reported phenol congener (436 cm^−1^).^30^ Among the indole hybrids, 8 (251 cm^−1^) showed a higher Stokes shift than 7 (210 cm^−1^). The optical bandgap of 6 (1.839 eV) was smaller than 4 (1.896 eV) and 5 (1.873 eV) among the pyrrole hybrids, and 8 (1.873) was smaller compared to 7 (1.896) in the indole series. Among the entire series of hybrids, the indole-containing hybrid 7 showed a longer fluorescence lifetime (9.91 ns) than the other hybrids (Table 2). The azopyrrole hybrid 4 (9.63 ns) and azoindole hybrid 7 (9.91) exhibited enhanced fluorescence lifetime, compared to their earlier reported arene analogs (9–9.31 ns).^30^ Conversely, the azo pyrrole hybrid 6 (5.76 ns) exhibited a shorter lifetime compared to its arene counterparts (6.56 to 7.04 ns).^30^

**Fig. 3:**
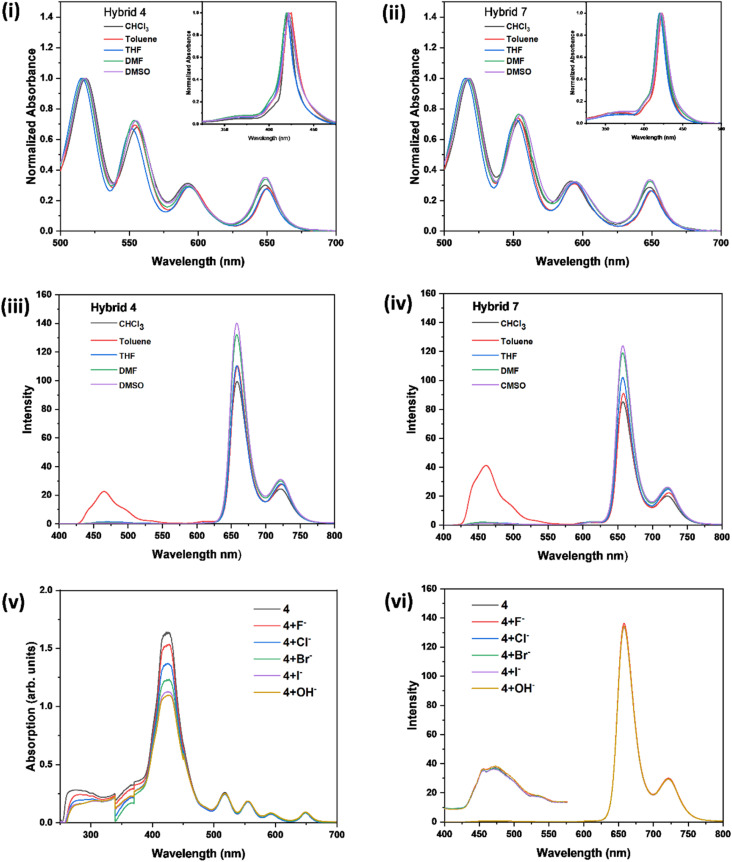
Comparison of (i) Q-band absorption spectra of hybrid 4 in different solvents (the inset shows their corresponding Soret band absorption spectra) (ii) Q-band absorption spectra of hybrid 7 in different solvents (the inset shows their corresponding Soret band absorption spectra) (iii) emission spectra of hybrid 4 in different solvents (iv) emission spectra of hybrid 7 in different solvents (v) absorption spectra of hybrid 4 after adding different anions (the changes in absorption values observed between 340-370 nm are due to the lamp change from UV to visible range and should be regarded as an instrumental response) (vi) emission spectra of hybrid 4 after adding different anions. All the spectra except the Soret bands shown in (i) & (ii) were recorded in 1 × 10^−5^ M concentration and, for the Soret-band measurement, the concentration used was 1 × 10^−6^ M.

To investigate the solvent effect, the absorption and emission spectra of hybrids 4 and 7 were measured in various solvents such as chloroform, tetrahydrofuran (THF), dimethylformamide (DMF), dimethyl sulfoxide (DMSO) in addition to toluene (Fig. 3i–iv). While no noticeable solvent-dependent shifts in the absorption or emission bands were observed, the emission intensities of both the hybrids were enhanced in DMF and DMSO compared to other solvents. Additionally, the emission corresponding to the π–π* transition of the azo-group (450–500 nm)^37^ was particularly pronounced in toluene (Fig. 3iii and iv).

The sensing ability of hybrids 4 and 7 towards various anions (F^−^, Cl^−^, Br^−^, I^−^, OH^−^) were investigated using UV–vis and fluorescence spectroscopic methods. The DMSO solutions of hybrids (1 × 10^−5^ M) and the aqueous solutions of either sodium or potassium salts of the respective anions (2 × 10^−3^ M) prepared in demineralized water was used for the studies. The intensity of the Soret band of hybrid 4 was quenched upon the addition of these anions, and the extent of quenching was significant in the case of I^−^ and OH^−^ (Fig. 3v). This could presumably be due to the formation of hydrogen bond between the pyrrolic NH and the I^−^ or due to the deprotonation of pyrrole NH, respectively.^38^ No significant changes were observed in the emission spectrum of hybrid 4 (Fig. 3vi) or in the absorption and emission spectra of the hybrid 7 upon the addition of these anions.

### Computational studies

**Fig. 4:**
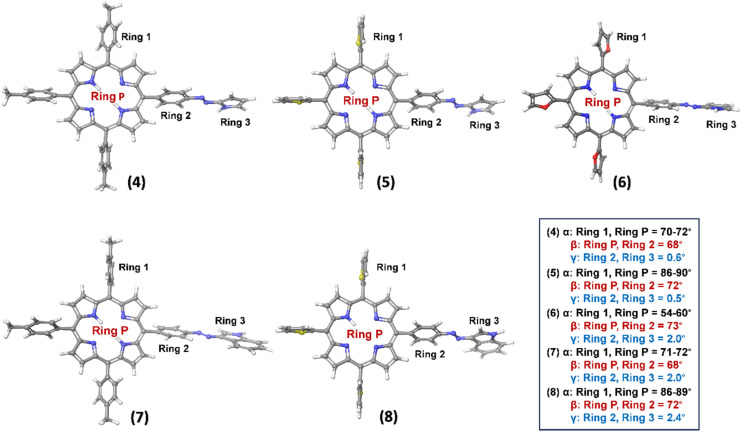
The optimized geometry of porphyrin-azoheteroarene hybrids 4–8. Selected dihedral angles α, β, γ are given in °.

Density functional theory (DFT) and time-dependent DFT (TD-DFT) calculations were performed using B3LYP functional theory and 6-311G** basis set to gain insights into the structural, spectral, and electronic properties of hybrids 4–8.^39–41^ The geometry-optimized structures of all the hybrids are shown in Fig. 4. Among the porphyrin-azopyrrole hybrids 4–6, the dihedral angle ‘α’ between ring 1 and the porphyrin plane (Ring P) in hybrid 6 was smaller compared to hybrids 4 and 5, indicating a minimal deviation of the *meso*-furyl groups from the mean porphyrin plane. The dihedral angle ‘β' between the porphyrin plane (Ring P) and the *meso*-aryl ring connected to the azo-bridge (Ring 2) was found to be approximately 70° in all hybrids, while the heteroarene moiety (Ring 3) was nearly coplanar with ring 2 (Table S1, SI).

**Fig. 5:**
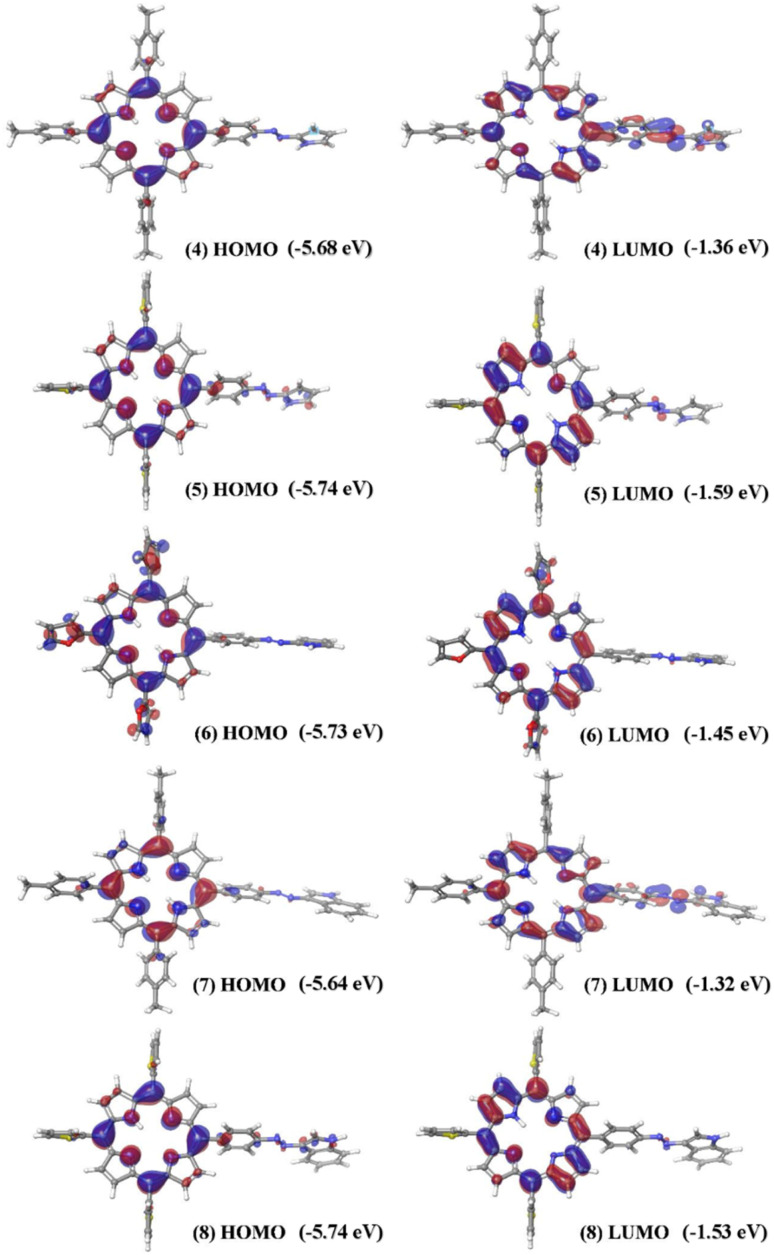
Representative sets of MOs of the porphyrin-azoheteroarene hybrids 4–8.

The HOMO and LUMO electron clouds were generally localized over the porphyrin ring in all the hybrids (Fig. 5). However, the porphyrin-centric frontier molecular orbital (FMO) characteristic was notably enhanced in hybrid 6, where the electron density extended over the three *meso*-furyl groups. In hybrids 4 and 7, the LUMO overlapped with the azoarene units, unlike the remaining hybrids that featured five-membered *meso*-substituents. This orbital overlap is attributed to the electron density drift from the macrocycle to the azo unit because of less efficient macrocyclic π-delocalization induced by the *meso-p*-tolyl groups compared to the thienyl and furyl groups. Furthermore, the theoretical calculations indicated that the band gap values for the *meso*-tris(2-thienyl/2-furyl) substituted hybrids 5, 6, and 8 were lower than those for the *meso*-tris(*p*-tolyl) substituted hybrids 4 and 7 (Table S1, SI).

The vertical excitation energies were calculated for all the hybrids based on their lowest energy conformers. In general, five absorption bands were deduced in the range of 407–590 nm for all the hybrids (Table S2, SI). The lowest energy absorption bands calculated for the *meso*-tris(2-furyl/2-thienyl) substituted porphyrin hybrids 5 (570 nm), 6 (590 nm), and 8 (580 nm) were of lower energies compared to the *meso*-(*p*-tolyl) substituted porphyrin-containing hybrids 4 (568 nm) and 7 (563 nm). Notably, hybrid 6, which contains the *meso*-tris(2-furyl) substituted porphyrin, showed the most red-shifted absorption at 590 nm, aligning well with the experimentally observed trend.

## Conclusion

This paper described the synthesis, photophysical, and computational studies of the first examples of porphyrin-containing hybrid molecules called porphyrin-azopyrroles and porphyrin-azoindoles. All the hybrids reported in this work exhibited higher fluorescence emission intensities than their precursor porphyrin, *i.e.*, the *meso*-(4-aminophenyl) substituted porphyrin. The *meso*-tris(2-furyl/2-thienyl) substituted porphyrin-containing hybrids exhibited red-shifted absorption/emission bands, more significant Stokes shifts, and smaller optical bandgaps than their counterparts containing all-*meso*-aryl-substituted porphyrins. The molar absorptivity coefficient values (*ε*) of all the absorption bands of porphyrin-azoindoles were higher than porphyrin-azopyrroles. The computational studies were in congruence with the experimental findings. The studies on the photoisomerization behaviour of these hybrids and their ramifications are currently underway in our laboratory.

## Experimental and computational methods

General chemicals, solvents, and silica gel were procured from indigenous brands like Spectrochem, Avra, and Loba Chemie and used without further purification unless otherwise specified. Freshly distilled pyrrole was used for porphyrin synthesis. *tert*-Butyl nitrite (^*t*^BuONO) was procured from Merck and used without further purifications. The 5-(4-aminophenyl)-10,15,20-tris(*p*-tolyl/2-thienyl/2-furyl) porphyrins 1, 2, and 3, respectively, were synthesized by following the procedures documented in the literature.

^1^H and ^13^C NMR spectra were recorded on Bruker instrument operating at 400 and 100.6 MHz respectively, by using tetramethylsilane [Si(CH_3_)_4_] as an internal standard. All the NMR measurements were carried out at room temperature in deuterated chloroform (CDCl_3_) solvent. The Mass spectrometric (MS) data were obtained using Agilent 6550 LC/Q-TOF and maXis impact 282 001.00081 instruments. The UV-visible and fluorescence measurements were carried out using Analytical Technologies Limited SPECTRO 2080+ and HITACHI F-4700 fluorescence spectrophotometer, respectively. The fluorescence quantum yields (*Φ*_f_) were estimated from the emission and absorption data by the comparative method using H_2_TPP (*Φ*_f_ = 0.11) as standard compound and the optical band gaps (*E*_opt_) were calculated by using wavelength corresponding to the intersection of normalized absorption and emission spectrum.^42,43^ Fluorescence lifetime measurements were performed by time-correlated single photon counting (TCSPC), using EDINBURGH instruments. Demineralized water was used for the anion sensing studies. Density functional theory (DFT) calculations were performed using Schrodinger's Material Science 3.9 package with the functional B3LYP in combination with the basis set 6-311G**. Optoelectronic calculations were carried out by using the adiabatic method (with Ag/AgCl as a reference electrode in inert condition).

### General synthesis of porphyrin-azopyrrole/indole hybrids 4–8

To an ice-cold solution of 5-(4-aminophenyl)-10,15,20-tris(*p*-tolyl/2-thienyl/2-furyl/)porphyrin 1 or 2 or 3 (75 μmol) in 20 mL of CH_3_CN/THF (1 : 1), ^*t*^BuONO (1.5 equiv., 11.6 mg, 13 μL) and a methanolic solution of either pyrrole (1.2 equiv., 6 mg, 6.2 μL) or indole (1.2 equiv., 10.4 mg) in pyridine were added slowly. The resulting solution was stirred at room temperature for 15 min. Then, the reaction mixture was concentrated under a vacuum, and the crude product was purified by silica gel chromatography using petroleum ether/dichloromethane (8 : 2 to 3 : 7, v/v) to obtain pure porphyrin-azopyrrole/indole hybrids 4–8 as purple solids.

#### Hybrid 4

(46 mg, 83%). M.p. >300 °C. ^1^H NMR (400 MHz, CDCl_3_): *δ* −2.74 (s, 2H, NH), 2.70 (s, 9H, CH_3_), 6.48–6.49 (m, 1H, pyrrole), 7.04–7.05 (m, 1H, pyrrole), 7.15–7.16 (m, 1H, pyrrole), 7.56 (d, *J* = 7.6 Hz, 6H, Ar), 8.10 (d, *J* = 7.6 Hz, 6H, Ar), 8.17 (d, *J* = 7.2 Hz, 2H, Ar), 8.31 (d, *J* = 7.2 Hz, 2H, Ar), 8.86–8.89 (m, 8H, pyrrole β-H) ppm. ^13^C{^1^H} NMR (100 MHz, CDCl_3_): *δ* 21.5, 111.8, 115.8, 119.0, 120.4, 121.9, 127.4, 131.1, 134.5, 135.4, 137.4, 139.2, 139.8, 143.7, 146.2, 152.1 ppm. ES MS C_51_H_40_N_7_: [M + H]^+^ Calc. mass 750.3340, found *m*/*z*: 750.3301.

#### Hybrid 5

(44 mg, 81%). M.p.>300 °C. ^1^H NMR (400 MHz, CDCl_3_): *δ* −2.64 (s, 2H, NH), 6.49 (t, *J* = 3.6 Hz, 1H, pyrrole), 7.05 (bs, 1H, pyrrole), 7.16 (d, *J* = 4.0 Hz, 1H, pyrrole), 7.50–7.51 (m, 3H, thienyl-H), 7.85–7.86 (m, 3H, thienyl-H), 7.92–7.93 (m, 3H, thienyl-H), 8.17 (d, *J* = 8.4 Hz, 2H, Ar), 8.30 (d, *J* = 8.4 Hz, 2H, Ar), 8.89 (d, *J* = 4.8 Hz, 2H, pyrrole β-H), 9.06 (s, 6H, pyrrole β-H) ppm. ^13^C{^1^H} NMR (100 MHz, CDCl_3_): *δ* 111.8, 111.9, 112.2, 115.9, 120.4, 120.7, 122.0, 126.1, 127.8, 127.9, 131.2, 133.8, 135.3, 142.7, 142.8, 143.1, 146.2, 152.3 ppm. LCMS (QTOF) C_42_H_27_N_7_S_3_: [M + H]^+^ Calc. mass 726.1586, found *m*/*z*: 726.1583.

#### Hybrid 6

(35 mg, 69%). M.p. >300 °C. ^1^H NMR (400 MHz, CDCl_3_): *δ* −2.60 (s, 2H, NH), 6.50 (bs, 1H, pyrrole), 7.03 (bs, 3H, furyl-H), 7.06 (bs, 1H, pyrrole), 7.17 (bs, 1H, pyrrole), 7.31–7.32 (m, 3H, furyl-H), 8.11 (bs, 3H, furyl-H), 8.19 (d, *J* = 8.0 Hz, 2H, Ar), 8.31 (d, *J* = 8.0 Hz, 2H, Ar), 8.92 (d, *J* = 4.0 Hz, 2H, pyrrole β-H), 9.13–9.18 (m, 6H, pyrrole β-H) ppm. ^13^C{^1^H} NMR (100 MHz, CDCl_3_): *δ* 108.6, 109.1, 111.8, 111.9, 116.0, 116.6, 116.7, 120.4, 121.0, 122.0, 123.5, 131.2, 135.5, 143.1, 144.2, 144.3, 146.2, 152.3, 154.2, 154.3 ppm. LCMS (QTOF) C_42_H_27_N_7_O_3_: [M + H]^+^ Calc. mass 678.2248, found *m*/*z*: 678.2247.

#### Hybrid 7

(11 mg, 18%). M.p. >300 °C. ^1^H NMR (400 MHz, CDCl_3_): *δ* −2.73 (s, 2H, NH), 2.70 (s, 9H, CH_3_), 7.38–7.40 (m, 2H, indole), 7.56 (d, *J* = 7.6 Hz, 6H, Ar), 8.07 (d, *J* = 2.8 Hz, 1H, indole), 8.10 (d, *J* = 7.6 Hz, 6H, Ar), 8.25 (d, *J* = 8.4 Hz, 2H, Ar), 8.35 (d, *J* = 8.4 Hz, 2H, Ar), 8.57 (bs, 1H, indole), 8.73–8.75 (m, 1H, indole), 8.70 (s, 4H, pyrrole β-H), 8.89 (d, *J* = 4.8 Hz, 2H, pyrrole β-H), 8.94 (d, *J* = 4.8 Hz, 2H, pyrrole β-H) ppm. ^13^C{^1^H} NMR (100 MHz, CDCl_3_): *δ* 21.5, 111.3, 119.0, 119.3, 120.0, 120.2, 120.3, 123.2, 123.3, 124.5, 127.4, 130.9, 135.3, 136.3, 137.3, 137.4, 139.2, 143.1, 153.2 ppm. LCMS (QTOF) C_55_H_41_N_7_: [M + H]^+^ Calc. mass 800.3496, found *m*/*z*: 800.3495.

#### Hybrid 8

(8 mg, 14%). M.p. >300 °C. ^1^H NMR (400 MHz, CDCl_3_): *δ* −2.63 (s, 2H, NH), 7.41–7.42 (m, 2H, indole), 7.49–7.51 (m, 3H, thienyl-H), 7.85–7.86 (m, 3H, thienyl-H), 7.92–7.93 (m, 3H, thienyl-H), 8.16 (d, *J* = 2.8 Hz, 1H, indole), 8.27 (d, *J* = 8.4 Hz, 2H, Ar), 8.34 (d, *J* = 8.4 Hz, 2H, Ar), 8.66 (bs, 1H, indole), 8.76 (d, *J* = 6.7 Hz, 1H, indole), 8.93 (d, *J* = 4.7 Hz, 2H, pyrrole β-H), 9.05 (s, 6H, pyrrole β-H) ppm. ^13^C{^1^H} NMR (100 MHz, CDCl_3_): *δ* 111.3, 111.7, 112.1, 118.9, 120.1, 121,0, 124.5, 126.0, 127.8, 131.0, 133.8, 135.3, 136.3, 137.3, 142.6, 142.7, 142.8, 153.4 ppm. LCMS (QTOF) C_46_H_29_N_7_S_3_: [M + H]^+^ Calc. mass 776.1717, found *m*/*z*: 776.1717.

## Author contributions

Sahana Nagesh Shet: investigation, validation, writing – original draft. Vighneshwar Ganesh Bhat: formal analysis. Swathi S. G.: investigation. Udaya Kumar Dalimba: validation, resources. Vijayendra S. Shetti: conceptualization, validation, supervision, project administration, funding acquisition, writing – original draft.

## Conflicts of interest

There are no conflicts to declare.

## Supplementary Material

RA-015-D5RA03790E-s001

## Data Availability

The data supporting this article have been included as part of the SI. Copies of ^1^H, ^13^C NMR spectra, HRMS, and cartesian coordinates of compounds 4–8 are available in SI. See DOI: https://doi.org/10.1039/d5ra03790e.
