# Supplementary material for: Porphyrin–azoheteroarenes: synthesis, photophysical, and computational studies
Source: RSC Adv. 2025 Aug 8;15(34):28240–7. doi: 10.1039/d5ra03790e (PMC12376762; doi:10.1039/d5ra03790e)
Supplement: RA-015-D5RA03790E-s001 [file RA-015-D5RA03790E-s001.pdf]

Supplementary Information (SI) for RSC Advances.
This journal is © The Royal Society of Chemistry 2025

Supporting Information

Porphyrin-azoheteroarenes: Synthesis, photophysical, and computational studies

Sahana Nagesh Shet,[a] Vighneshwar Ganesh Bhat,[a] Swathi S. G.,[a] Udaya Kumar Dalimba,[a] Vijayendra S. Shetti [a*]

[a] Department of Chemistry, National Institute of Technology Karnataka, Surathkal-575025, India

==

Contents	Page no.
**Figure S1**. ^{1}H NMR spectrum of **4**	S2
**Figure S2**. ^{13}C NMR spectrum of **4**	S3
**Figure S3**. ES MS spectrum of **4**	S4
**Figure S4**. ^{1}H NMR spectrum of **5**	S5
**Figure S5**. ^{13}C NMR spectrum of **5**	S6
**Figure S6**. LCMS QTOF spectrum of **5**	S7
**Figure S7**. ^{1}H NMR spectrum of **6**	S8
**Figure S8**. ^{13}C NMR spectrum of **6**	S9
**Figure S9**. LCMS QTOF spectrum of **6**	S10
**Figure S10**. ^{1}H NMR spectrum of **7**	S11
**Figure S11**. ^{13}C NMR spectrum of **7**	S12
**Figure S12**. LCMS QTOF spectrum of **7**	S13
**Figure S13**. ^{1}H NMR spectrum of **8**	S14
**Figure S14**. ^{13}C NMR spectrum of **8**	S15
**Figure S15**. LCMS QTOF spectrum of **8**	S16
**Figure S16**. Fluorescence decay profiles of hybrids **4-8**	S17
**Figure S17**. Excitation spectra of hybrids **4** and **7**	S18
**Table S1**. The selected dihedral angles (in °) and energies in the lowest energy conformers of porphyrin-azoheteroarenes	S19
**Table S2**. Calculated vertical excitation (nm) and oscillator strength (f) for the porphyrin-azoheteroarenes	S19
**Table S3**. Optimized Geometries (Cartesian coordinates) of the porphyrin-azoheteroarenes	S19

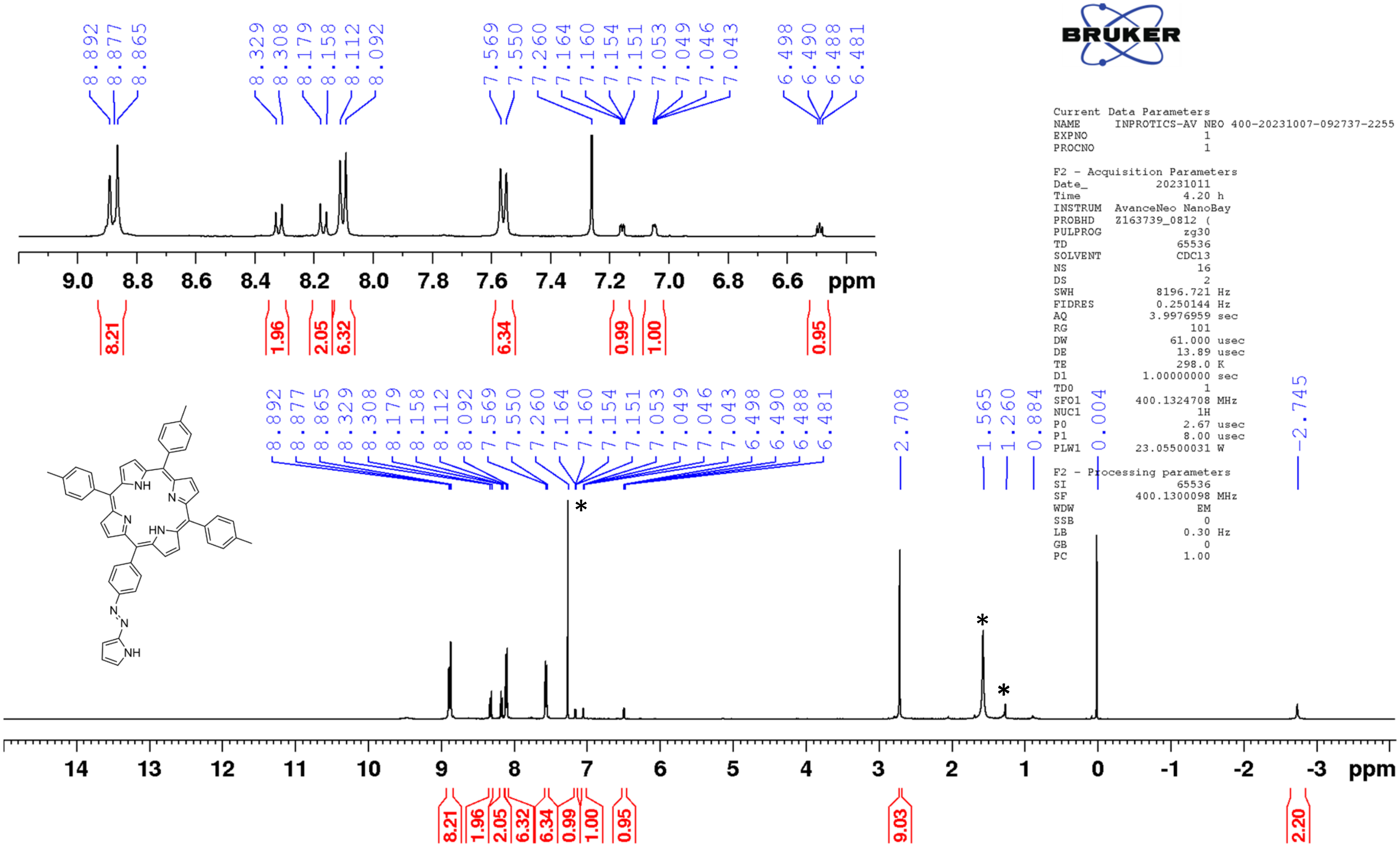

Figure S1. ^{1}H NMR spectrum of **4** (* indicates residual solvent peaks)

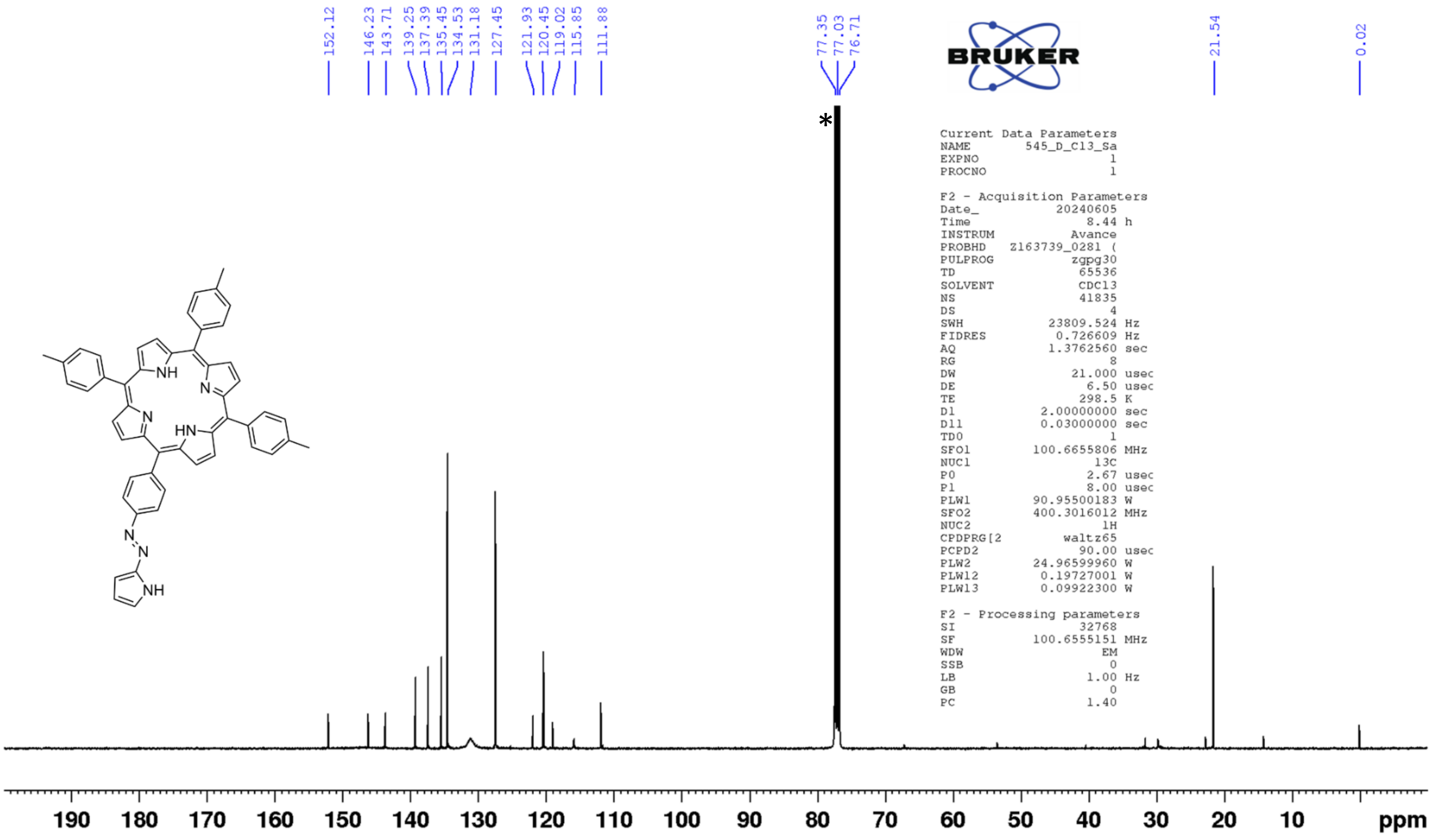

Figure S2. ^{13}C NMR spectrum of **4** (* indicates residual solvent peaks)

DEPARTMENT OF CHEMISTRY, I.I.T.(B)

Analysis Info

Analysis Name	D:\Data\JUNE-24\MR-SB-VSSSNS30.d	Acquisition Date	6/17/2024 11:54:19 AM
Method	Naformat_pos_1000.m	Operator	ssk-in
Sample Name	MR-SB-VSSSNS30	Instrument	maXis impact 282001.00081
Comment	C51H39N7		

Acquisition Parameter

Source Type	ESI	Ion Polarity	Positive	Set Nebulizer	0.3 Bar
Focus	Not active	Set Capillary	3700 V	Set Dry Heater	180 °C
Scan Begin	50 m/z	Set End Plate Offset	-500 V	Set Dry Gas	4.0 l/min
Scan End	1000 m/z	Set Charging Voltage	2000 V	Set Divert Valve	Source
		Set Corona	0 nA	Set APCI Heater	0 °C

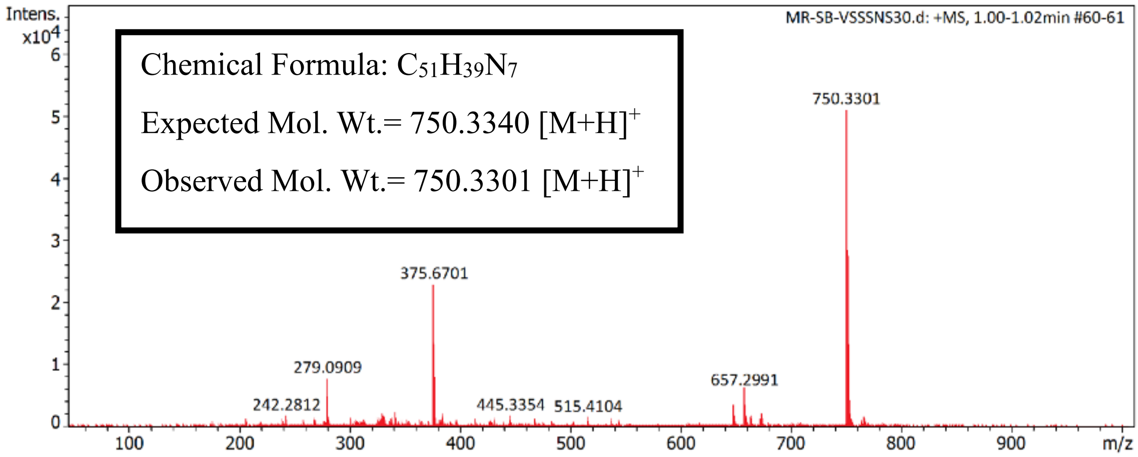

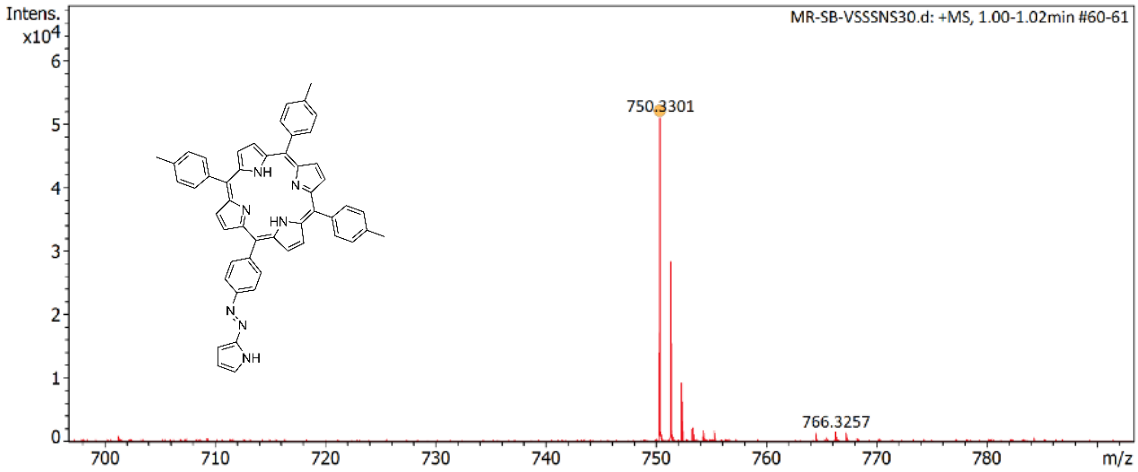

Meas. m/z	#	Ion Formula	m/z	err [ppm]	mSigma	# mSigma	Score	rdb	e⁻ Conf	N-Rule
750.3301	1	C51H40N7	750.3340	5.2	34.7	2	100.00	36.0	even	ok

MR-SB-VSSSNS30.d
Bruker Compass DataAnalysis 5.1 printed: 6/18/2024 10:19:08 AM by: ssk-in Page 1 of 1

Figure S3. ES MS spectrum of **4**

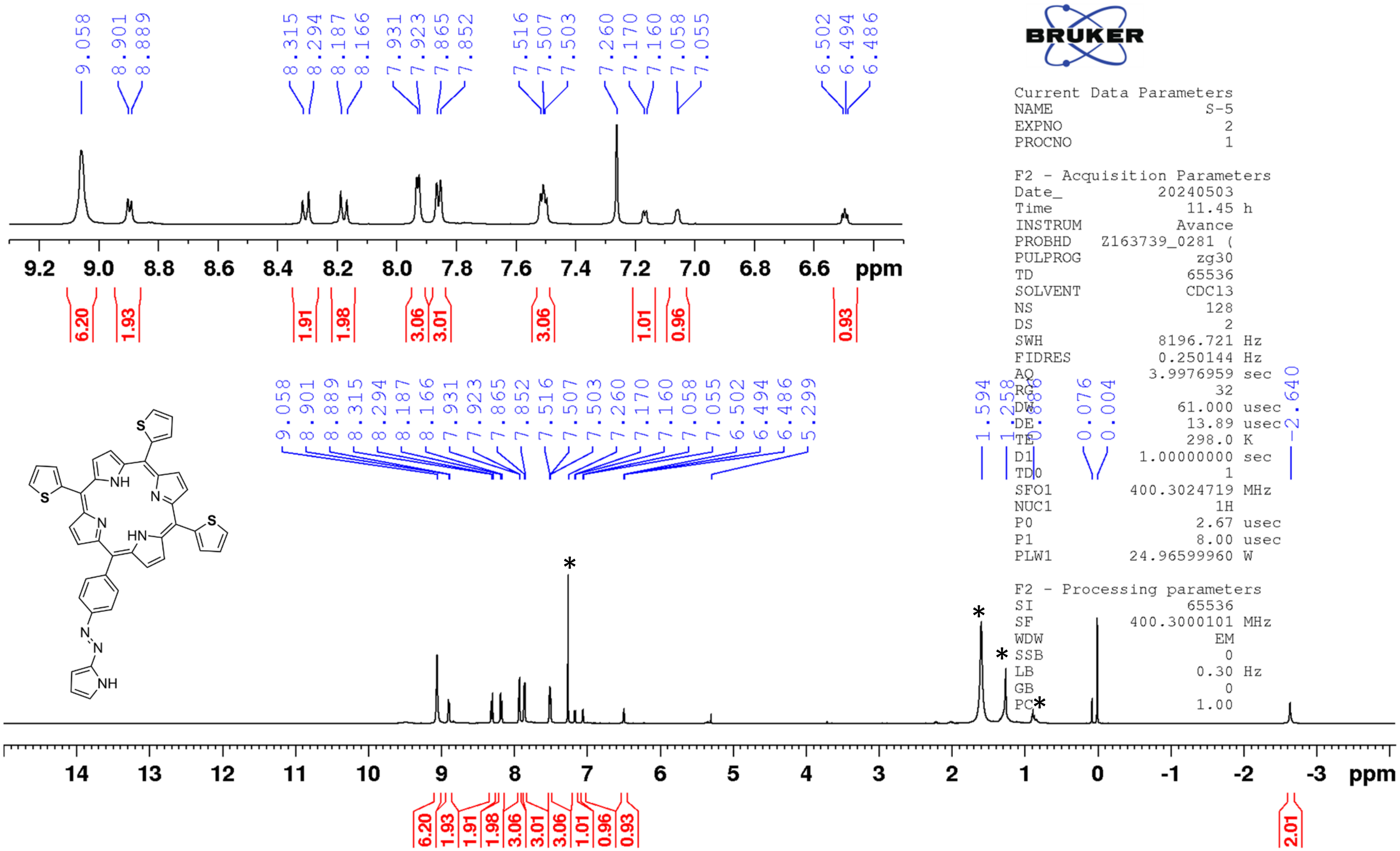

Figure S4. ^{1}H NMR spectrum of **5** (* indicates residual solvent peaks)

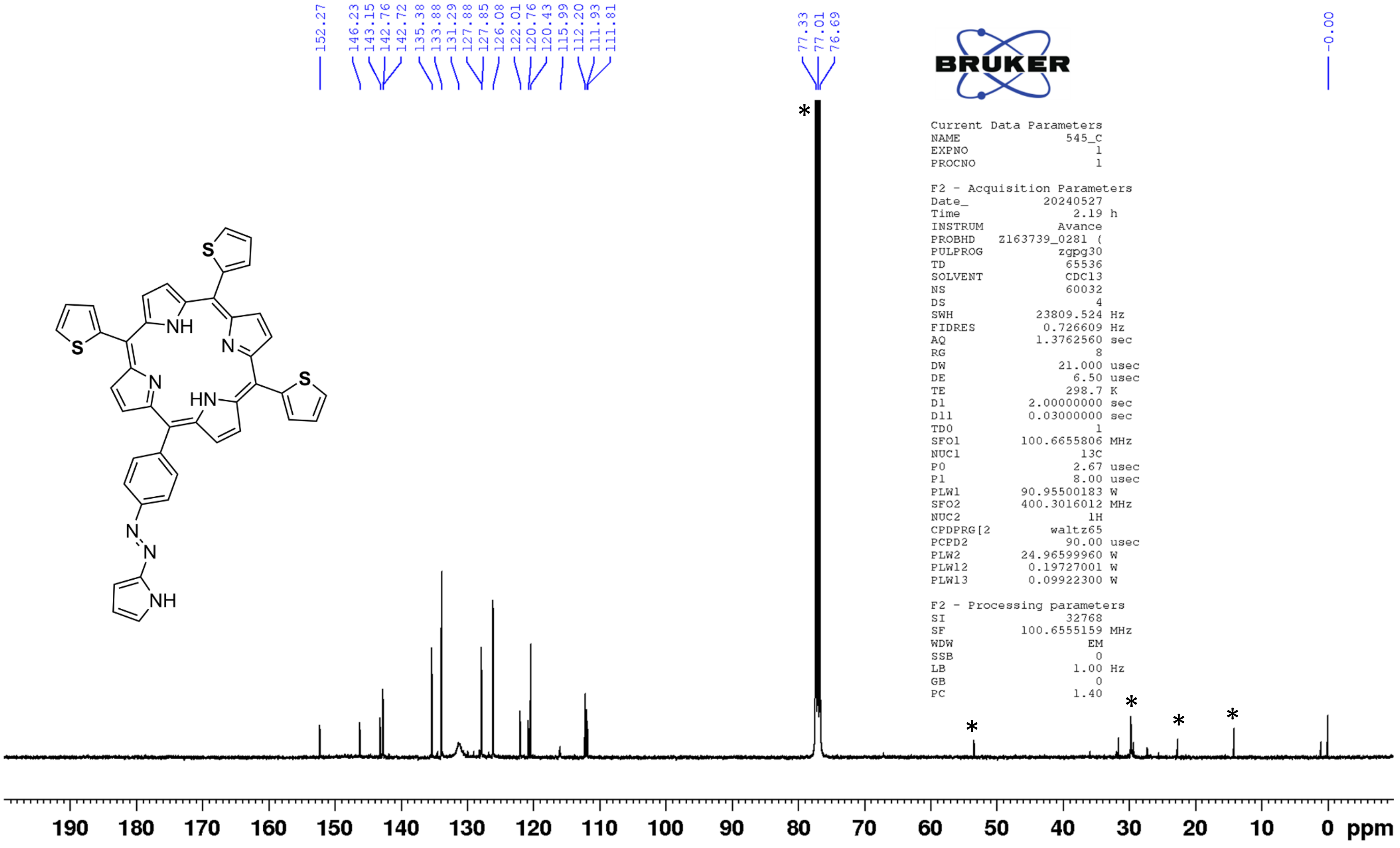

Figure S5. ^{13}C NMR spectrum of **5** (* indicates residual solvent peaks)

Sample Information

**Name**	MR-SB-SNS-31	**Data File Path**	X:\Projects\MASS Data\Data\MAY-24\MR-SB-SNS-31.d
**Sample ID**		**Acq. Time (Local)**	5/27/2024 12:00:44 PM (UTC+05:30)
**Instrument**	LCMSQTOF-G6545B	**Method Path (Acq)**	D:\Projects\MASS Data\Methods\A1B1_POS_100-1000_4000_500_120.m
**MS Type**	QTOF	**Version (Acq SW)**	6200 series TOF/6500 series Q-TOF (11.0.203.0)
**Inj. Vol. (ul)**	0.3	**IRM Status**	Success
**Position**	P2-B2	**Method Path (DA)**	D:\MassHunter\Report Templates\REPORT METHOD\HRMS_IITB_1.m
**Plate Pos.**		**Target Source Path**	
**Operator**	SYSTEM (SYSTEM)	**Result Summary**	1 qualified (1 targets)

Sample Spectra

+ Scan (rt: 0.246-0.379 min)

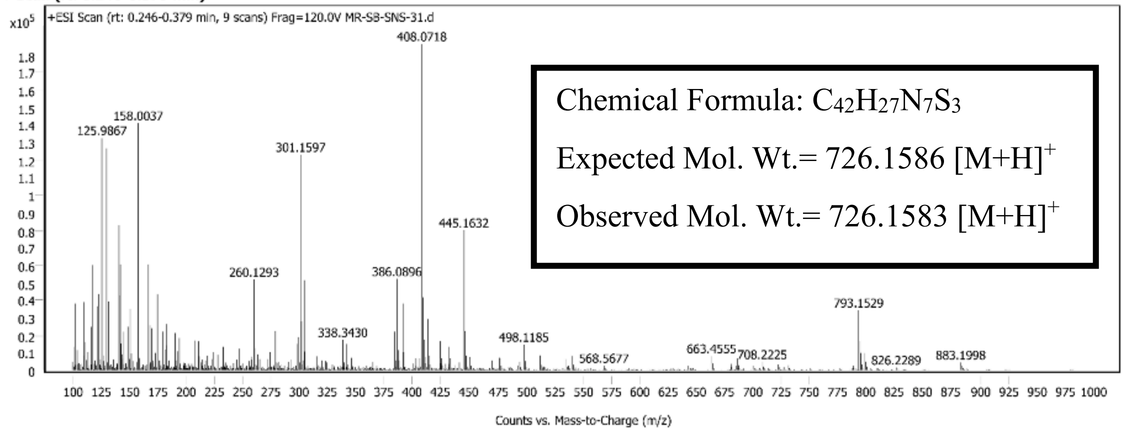

Compound Details

Cpd. 1: C42 H27 N7 S3

Formula	m/z	Observed M/Z	Difference Da	Difference PPM	Score
C42 H27 N7 S3	726.1583	726.158302557461	2.05251770250925	2.83047716432701	96.54

Compound Spectra (Zoomed)

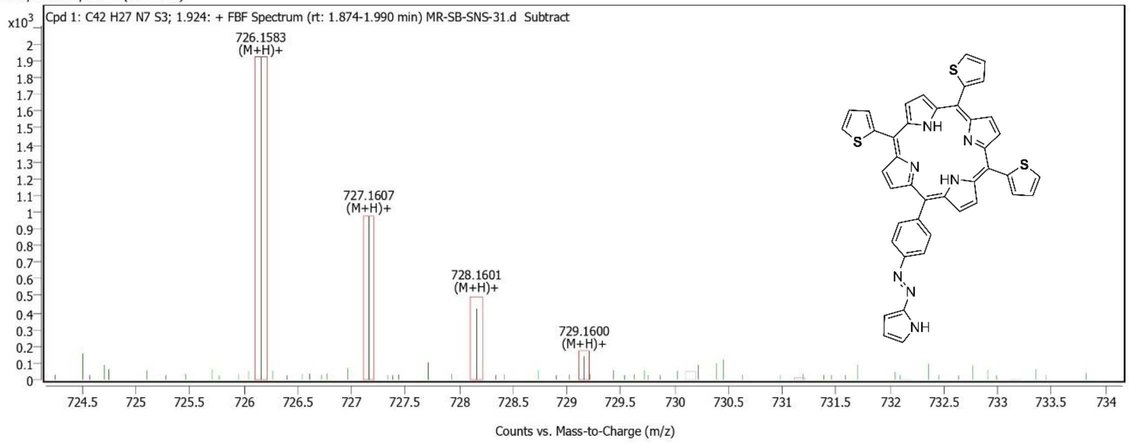

MassHunter Qual 10.0
(End of Report)

Figure S6. LCMS QTOF spectrum of **5**

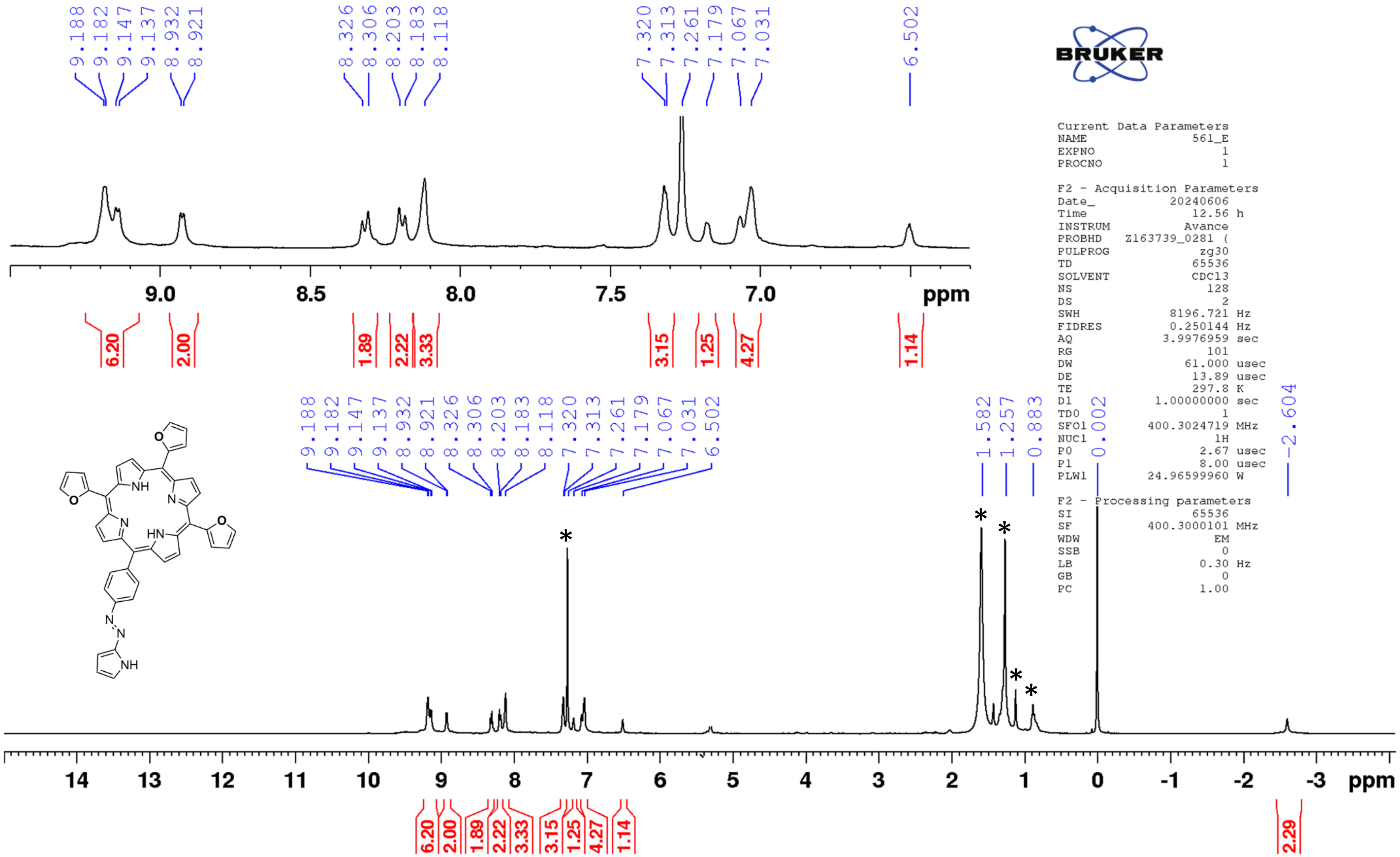

Figure S7. ^{1}H NMR spectrum of **6** (* indicates residual solvent peaks)

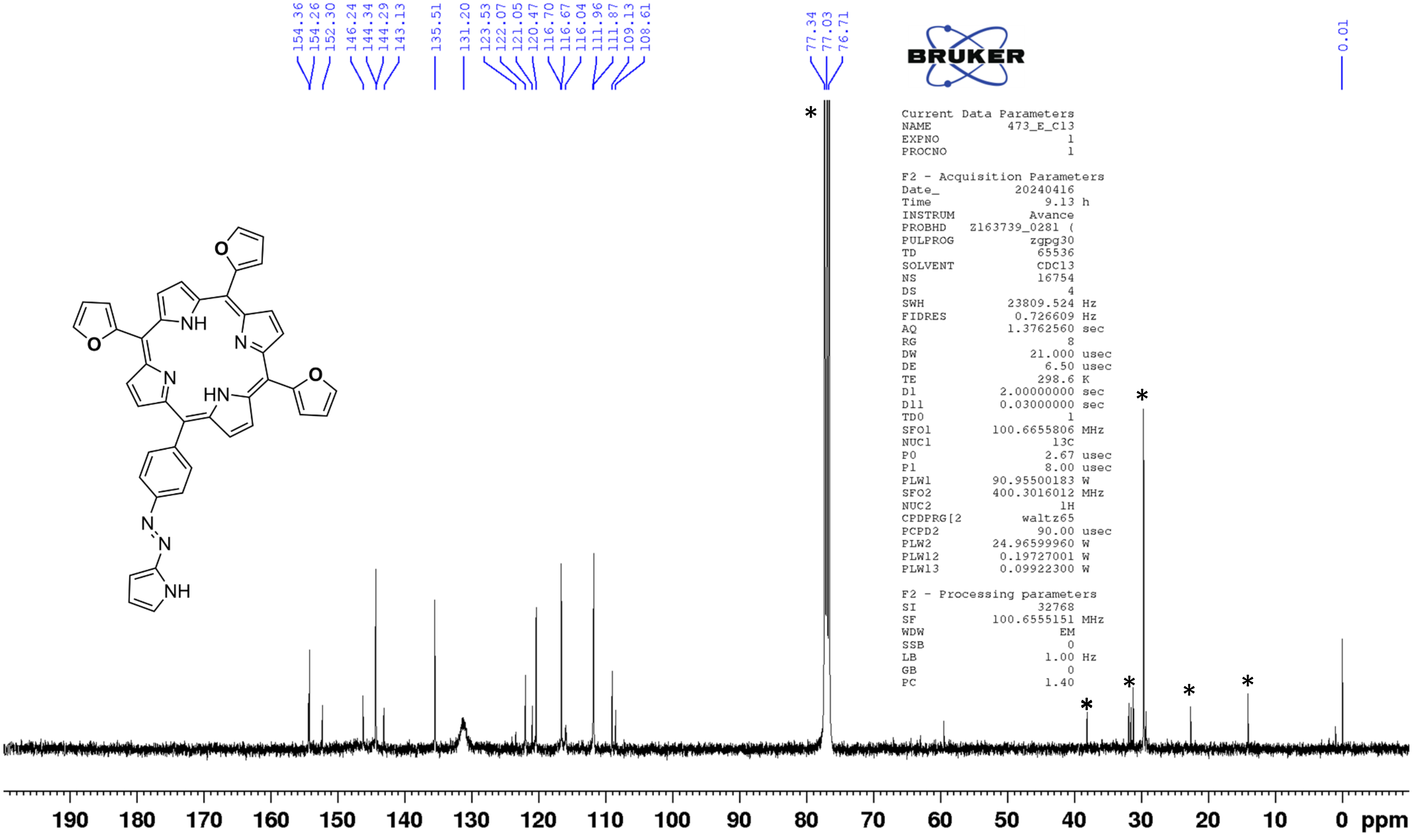

Figure S8. ^{13}C NMR spectrum of **6** (* indicates residual solvent peaks)

Department of Chemistry I.I.T. (B)

Agilent Trusted Answers

Sample Information

**Name**	MR-SB-VSS-SNS-32	**Data File Path**	X:\Projects\MASS Data\Data\MAY-24\MR-SB-VSS-SNS-32.d
**Sample ID**		**Acq. Time (Local)**	5/30/2024 12:09:32 PM (UTC+05:30)
**Instrument**	LCMSQTOF-G6545B	**Method Path (Acq)**	D:\Projects\MASS Data\Methods\A1B1_POS_100-1500_4000_800_220.m
**MS Type**	QTOF	**Version (Acq SW)**	6200 series TOF/6500 series Q-TOF (11.0.203.0)
**Inj. Vol. (ul)**	0.5	**IRM Status**	Success
**Position**	P1B1	**Method Path (DA)**	D:\MassHunter\Report Templates\REPORT METHOD\HRMS_IITB_1.m
**Plate Pos.**		**Target Source Path**	
**Operator**	SYSTEM (SYSTEM)	**Result Summary**	1 qualified (1 targets)

Sample Spectra

+ Scan (rt: 0.179-0.629 min) **Peak 1 from + TIC Scan**

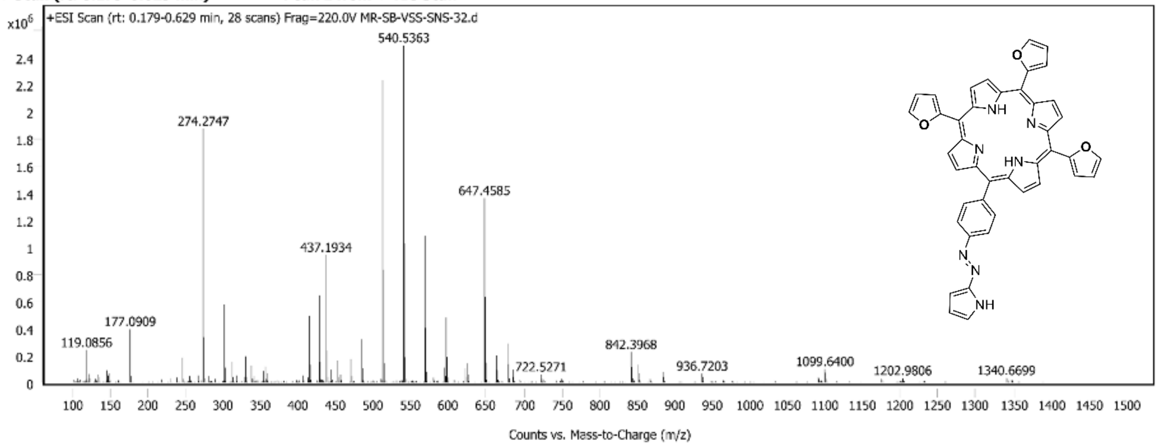

Compound Details

Cpd. 1: C42 H27 N7 O3

Formula	m/z	Observed M/Z	Difference_Da	Difference_PPM	Score
C42 H27 N7 O3	678.2248	678.224752295974	-0.135014711645454	-0.199366826925434	99.91

Compound Spectra (Zoomed)

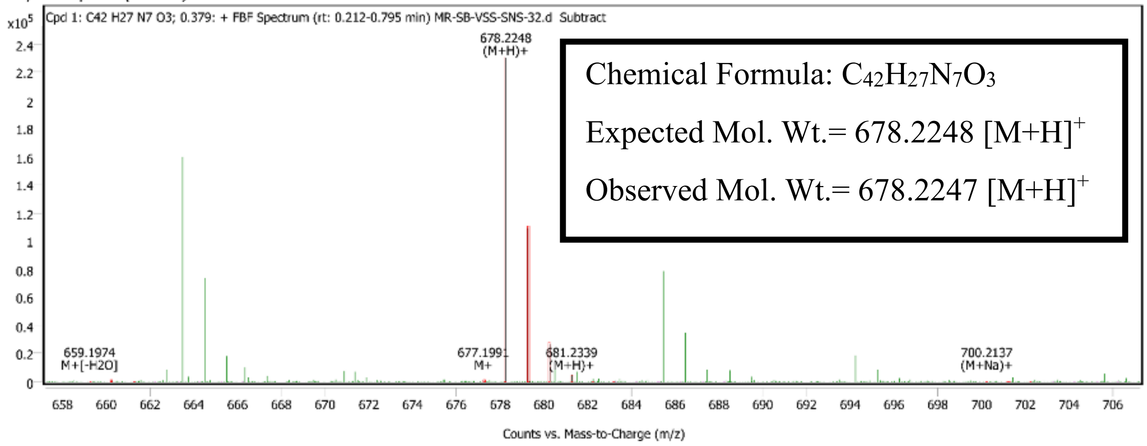

MassHunter Qual 10.0
(End of Report)

Figure S9. LCMS QTOF spectrum of **6**

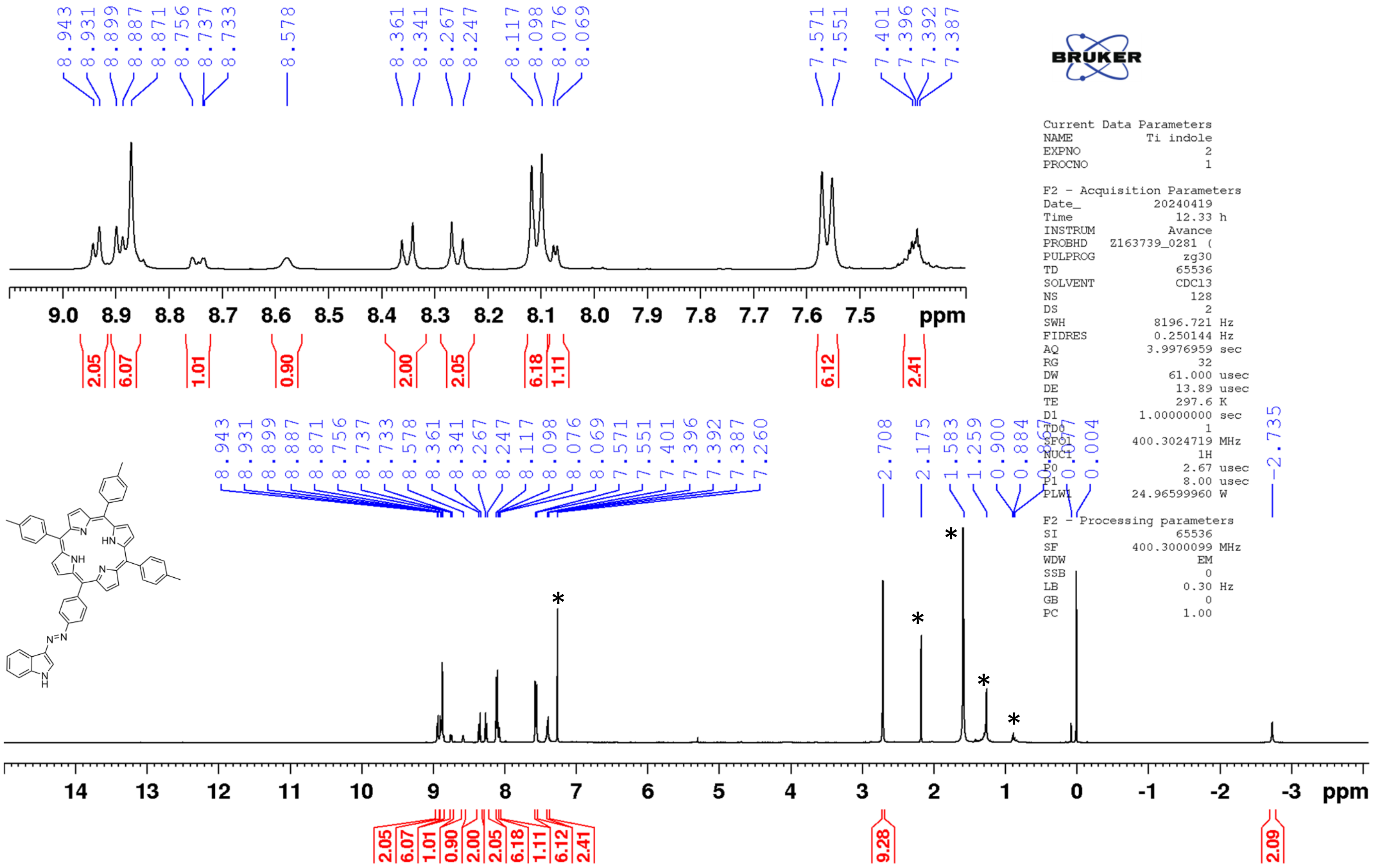

Figure S10. ^{1}H NMR spectrum of **7** (* indicates residual solvent peaks)

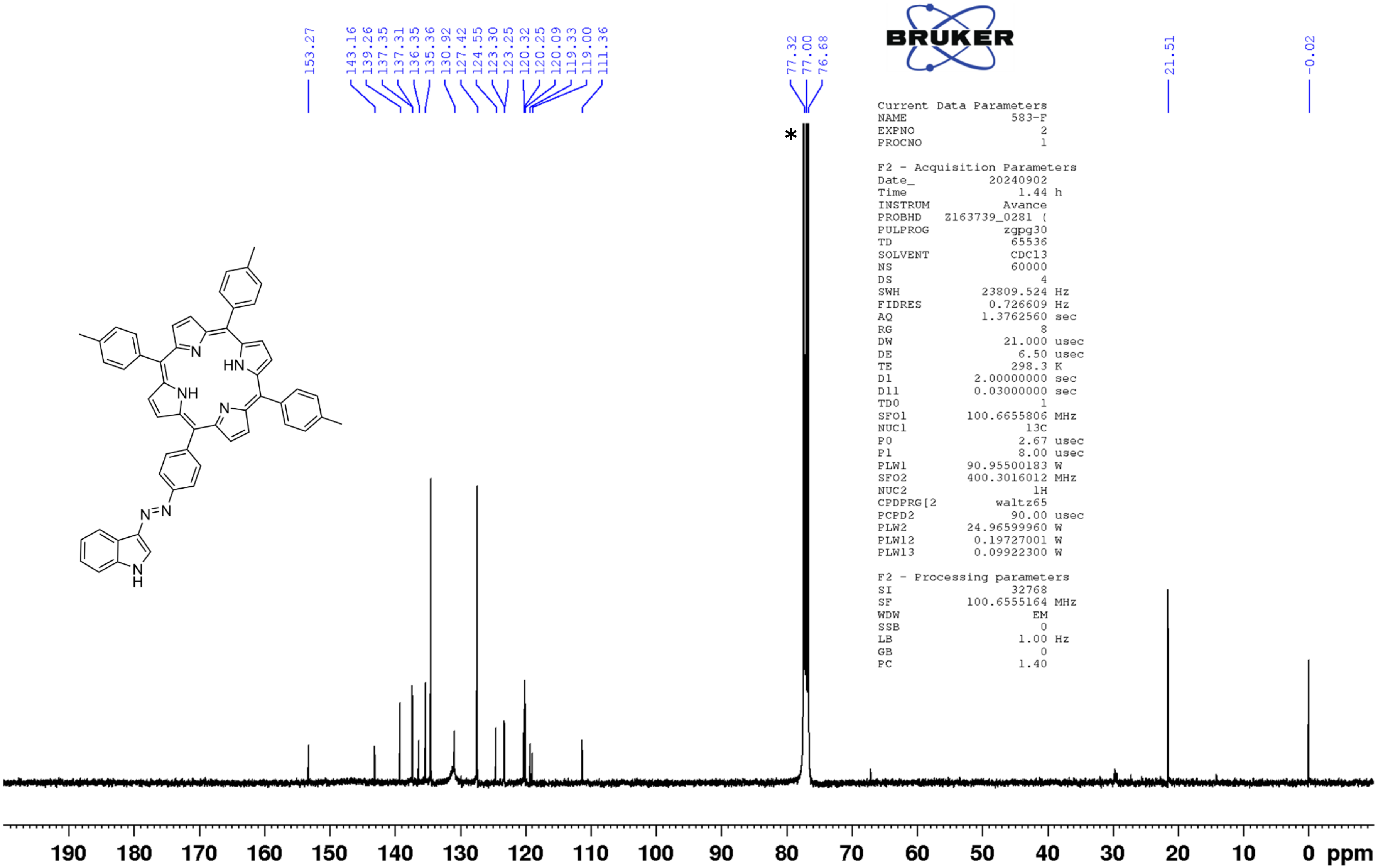

Figure S11. ^{13}C NMR spectrum of **7** (* indicates residual solvent peaks)

Department of Chemistry I.I.T. (B)

Agilent | Trusted Answers

Sample Information

**Name**	MR-SB-VSS-SNS-33	**Data File Path**	X:\Projects\MASS Data\Data\MAY-24\MR-SB-VSS-SNS-33.d
**Sample ID**		**Acq. Time (Local)**	5/30/2024 12:25:55 PM (UTC+05:30)
**Instrument**	LCMSQTOF-G6545B	**Method Path (Acq)**	D:\Projects\MASS Data\Methods\A1B1_POS_100-1500_4000_800_220.m
**MS Type**	QTOF	**Version (Acq SW)**	6200 series TOF/6500 series Q-TOF (11.0.203.0)
**Inj. Vol. (ul)**	0.5	**IRM Status**	Success
**Position**	P1B1	**Method Path (DA)**	D:\MassHunter\Report Templates\REPORT METHOD\HRMS_IITB_1.m
**Plate Pos.**		**Target Source Path**	
**Operator**	SYSTEM (SYSTEM)	**Result Summary**	1 qualified (1 targets)

Sample Spectra

+ Scan (rt: 0.215-0.598 min) **Peak 1 from + TIC Scan**

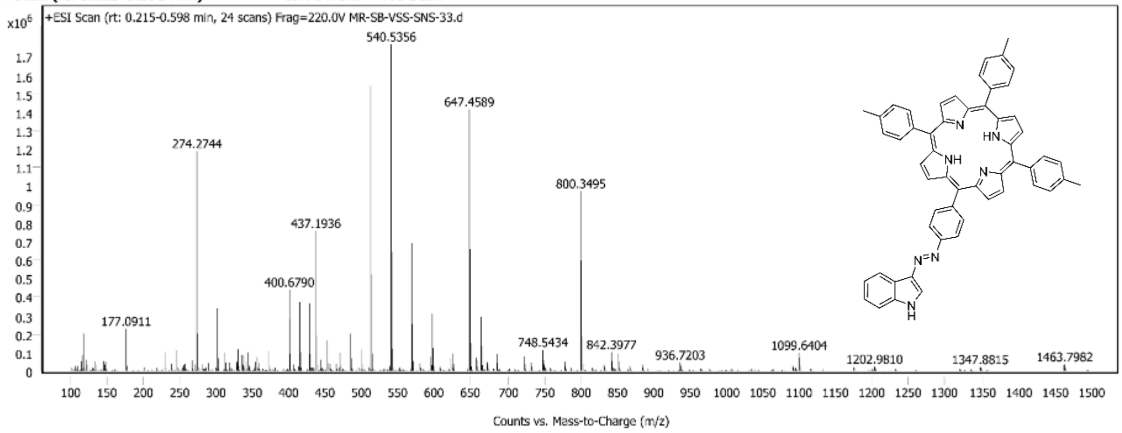

Compound Details

Cpd. 1: C55 H41 N7

Formula	m/z	Observed M/Z	Difference_Da	Difference_PPM	Score
C55 H41 N7	800.3496	800.349585686503	-0.0126728011764499	-0.0158540345896491	99.92

Compound Spectra (Zoomed)

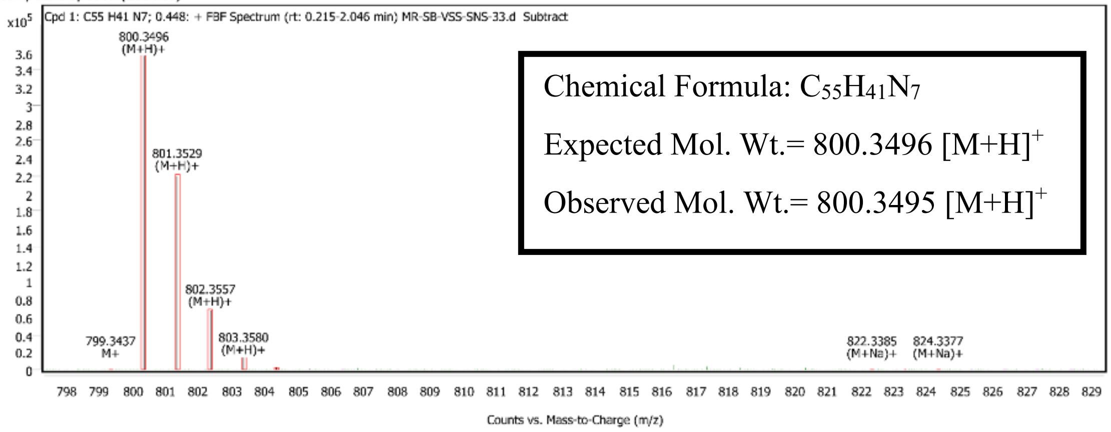

MassHunter Qual 10.0
(End of Report)

MassHunter Qualitative Analysis Page 1 of 1 Generated at 12:38 PM on 5/30/2024

Figure S12. LCMS QTOF spectrum of **7**

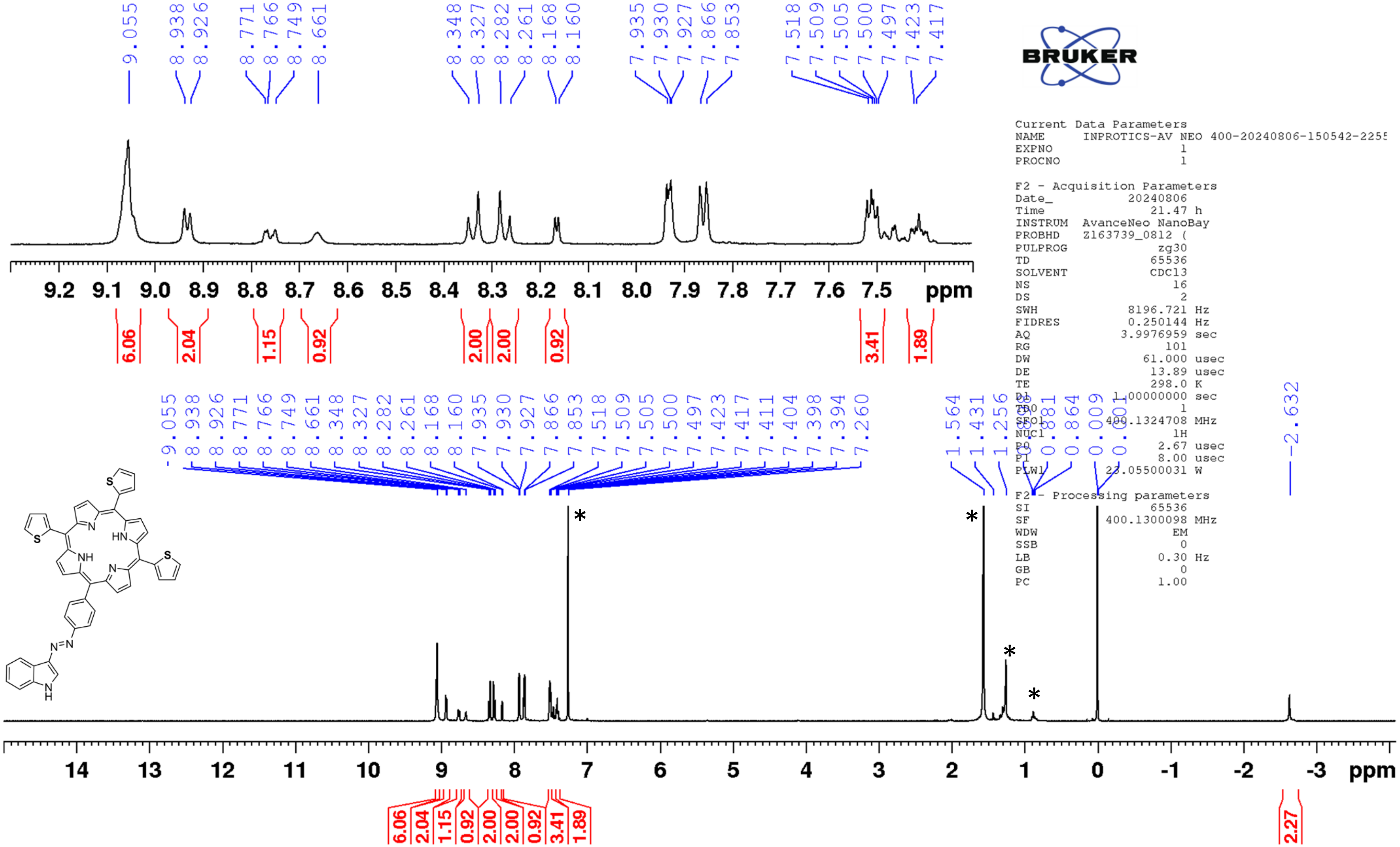

Figure S13. ^{1}H NMR spectrum of **8** (* indicates residual solvent peaks)

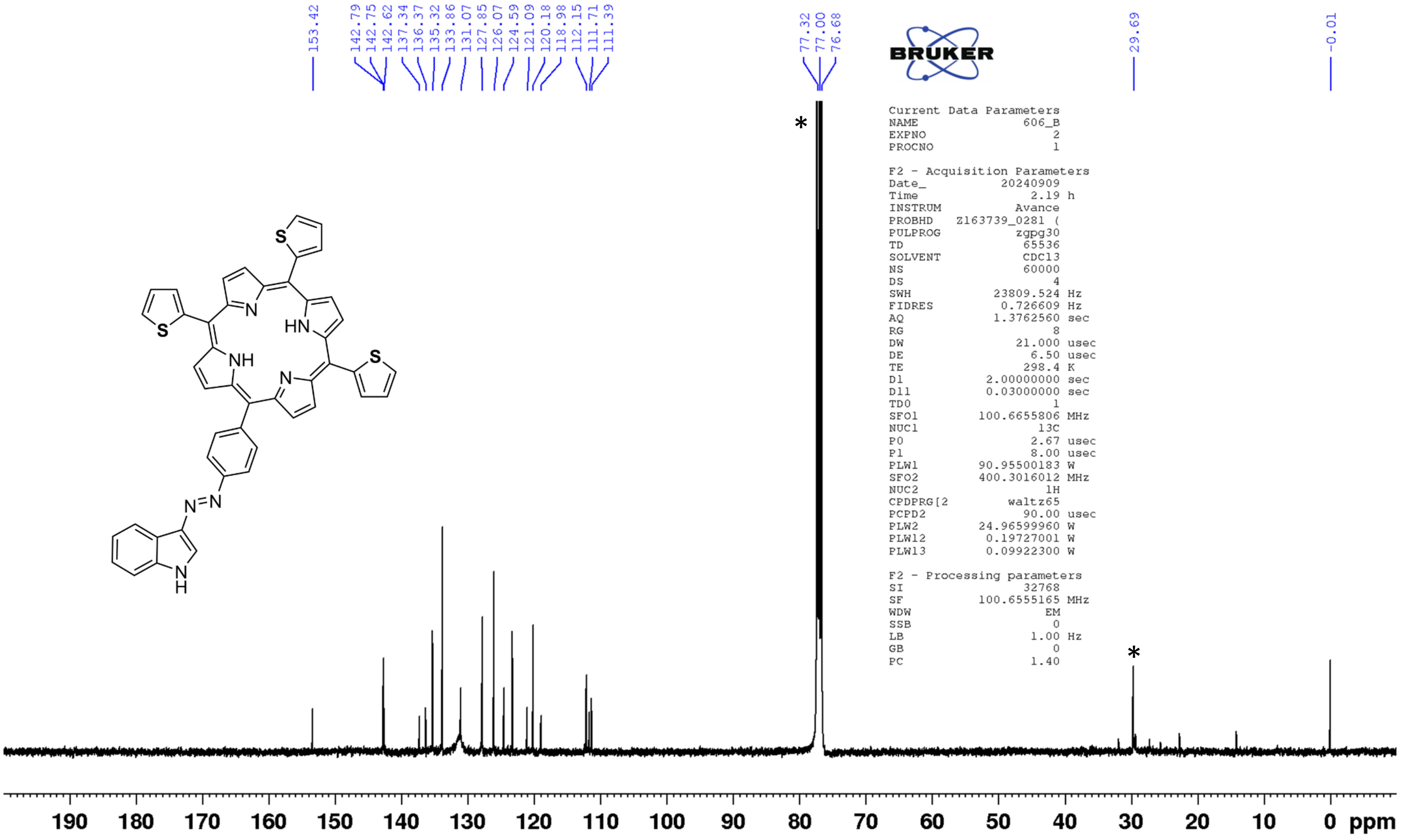

Figure S14. ^{13}C NMR spectrum of **8** (* indicates residual solvent peaks)

Department of Chemistry I.I.T. (B)

Agilent | Trusted Answers

Sample Information

**Name**	MR-SB-VSS-SNS-40	**Data File Path**	D:\Projects\MASS Data\Data\JULY-2024\MR-SB-VSS-SNS-40.d
**Sample ID**		**Acq. Time (Local)**	29-07-2024 12:13:08 (UTC+05:30)
**Instrument**	LCMSQTOF-G6545B	**Method Path (Acq)**	D:\Projects\MASS Data\Methods\A1B1_POS_100-1000_4000_500_120.m
**MS Type**	QTOF	**Version (Acq SW)**	6200 series TOF/6500 series Q-TOF (11.0.203.0)
**Inj. Vol. (ul)**	6	**IRM Status**	Success
**Position**	P2A2	**Method Path (DA)**	C:\Users\LCMS QTOF G6545\Desktop\Report Templates\REPORT METHOD\HRMS_1.m
**Plate Pos.**		**Target Source Path**	
**Operator**	SYSTEM (SYSTEM)	**Result Summary**	1 qualified (1 targets)

Sample Spectra

+ Scan (rt: 0.224-0.440 min) **Peak 1 from + TIC Scan**

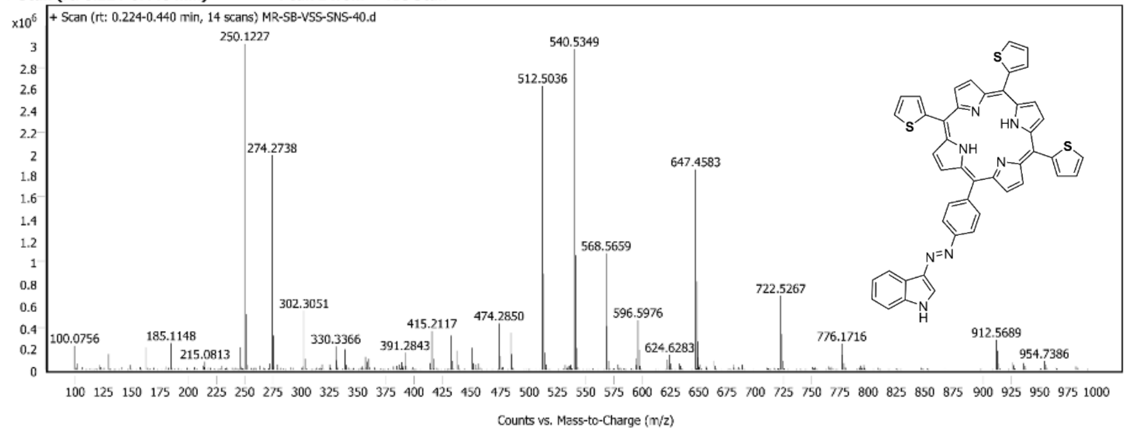

Compound Details

Cpd. 1: C46 H29 N7 S3

Formula	m/z	Observed M/Z	Difference Da	Difference PPM	Score
C46 H29 N7 S3	776.1717	776.171721564506	-0.414049498772329	-0.534143933780154	98.63

Compound Spectra (Zoomed)

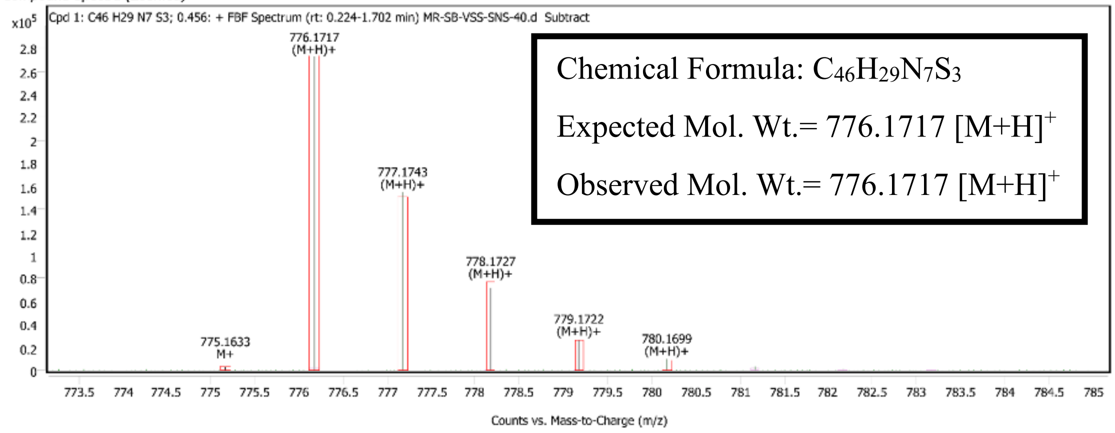

MassHunter Qual 10.0
(End of Report)

Figure S15. LCMS QTOF spectrum of **8**

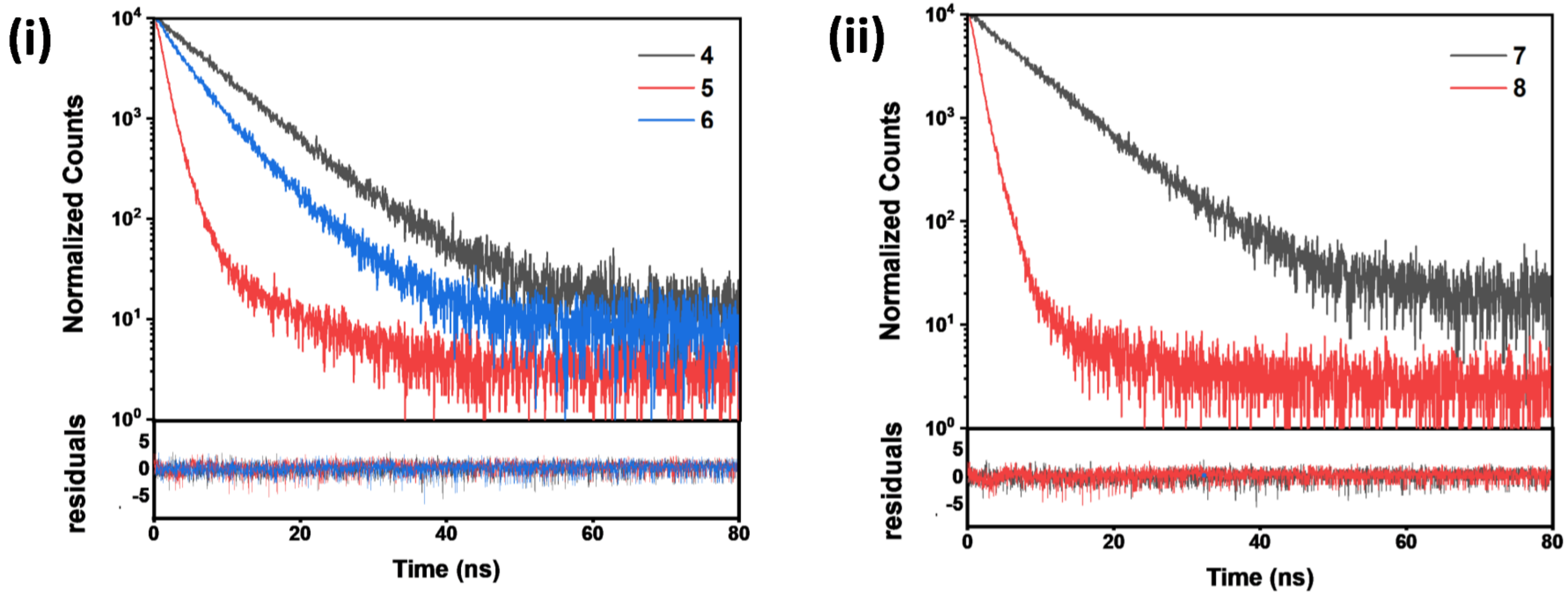

Figure S16. (i) Fluorescence decay profile of hybrids **4** (black line), **5** (red line), and **6** (blue line), (ii) fluorescence decay profile of hybrids **7** (black line) and **8** (red line). All the spectra were recorded in $1{\times}10^{-5}$ M solution of toluene.

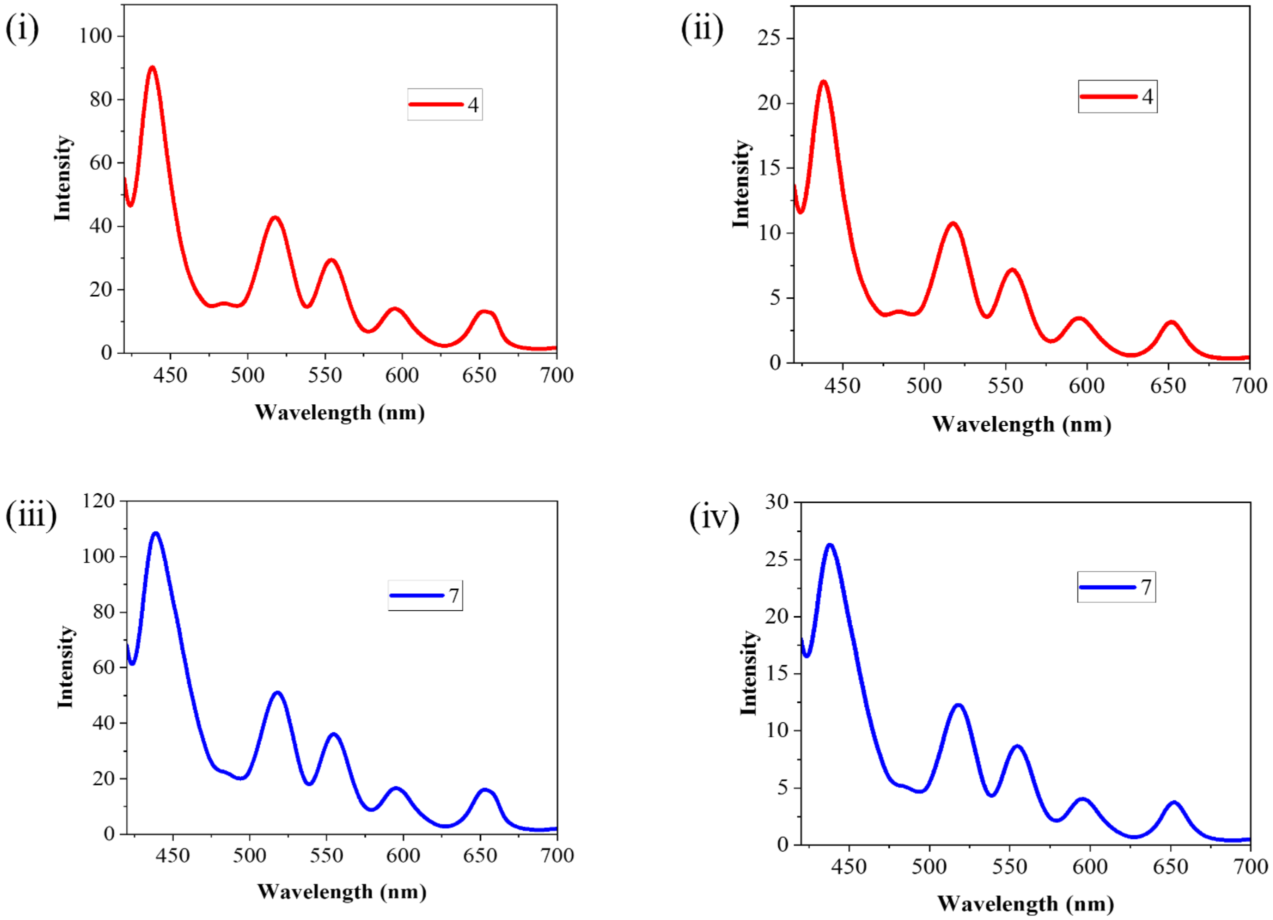

Figure S17. i) Excitation spectra of hybrid i) **4** ($\lambda_{em.}$ = 660 nm), ii) **4** (λ_{em} = 725 nm), iii) **7** (λ_{em} = 660 nm), iv) **7** (λ_{em} = 725 nm). All the spectra were recorded in 1×10^{-5} M solution of toluene.

Table S1. The selected dihedral angles (in °) and energies in the lowest energy conformers of the porphyrin-azoheteroarenes

Compound	α in °	β in °	γ in °	HOMO (eV)	LUMO (eV)	Band gap (eV)
**4**	70-72	68	0.6	-5.68	-1.36	4.31
**5**	86-90	72	0.5	-5.74	-1.59	4.14
**6**	54-60	73	2.0	-5.73	-1.45	4.28
**7**	71-72	68	2.0	-5.64	-1.32	4.32
**8**	86-89	72	2.4	-5.74	-1.35	4.20

Table S2. Calculated vertical excitation (nm) and oscillator strength (*f*) for the porphyrin-azoheteroarenes

Compound	λ (nm), Oscillator strength(*f*)
**4**	407.8 (0.51), 429.7 (0.00), 475.2 (0.16), 522.8 (0.16), 567.6 (0.076)
**5**	428.5 (0.01), 429.7 (0.176), 439.3 (0.15), 528.4 (0.14), 570.0 (0.045)
**6**	425.0 (0.09), 427.2 (0.001), 466.1 (0.095), 540.5 (0.22), 590.2 (0.057)
**7**	414.1 (0.44), 441.0 (0.001), 450.5 (0.18), 518.7 (0.14), 563.4 (0.003)
**8**	439.9 (0.23), 443.6 (0.01), 444.0 (0.037), 538.6 (0.184), 579.7 (0.07)

Table S3. Optimized Geometries (Cartesian coordinates) of the porphyrin-azoheteroarenes

Optimized geometries (Cartesian coordinates in Å) and energies of porphyrin-arene hybrids (in eV). Notation: E = gas phase energy, Nimag = number of imaginary frequencies.

**4**<br>E = -2350.6940<br>Nimag = 0				**5**<br>E = -3194.9985<br>Nimag = 0			
C1	-2.9109922091	5.6163451410	1.0601519971	C1	-3.4500479349	3.3018004264	0.1595869478
C2	-3.3423026327	4.8900800286	-0.0574458117	C2	-4.7090419946	2.6911717608	0.0939405652
C3	-2.8713039511	7.0057731131	1.0364625099	C3	-2.2119168515	2.6245632288	0.1885208144
C4	-3.2559318806	7.7229559650	-0.1023166736	C4	-5.9820243311	3.3485027430	0.0396119877
C5	-3.6849759617	6.9966597051	-1.2150473759	C5	-6.9524275104	2.3902660056	-0.0375805795
C6	-3.7307001003	5.6041301158	-1.1946984857	C6	-6.3134054001	1.1068371683	-0.0281032366
C7	-3.3854587453	3.3929650094	-0.0326560603	N7	-4.9632845488	1.3416651946	0.0523301081
C8	-4.6577450577	2.8028498222	-0.0209001500	N8	-2.0348840156	1.2748066765	0.2082873439
C9	-2.1632887369	2.6915654252	-0.0178433210	C9	-0.6838162338	1.0824293538	0.2415289814
C10	-5.9189569066	3.4801502908	0.0370935179	C10	0.0208631589	2.3605079823	0.2220561799
C11	-6.9099515024	2.5381933215	0.0298871075	C11	-0.9303044365	3.3193984627	0.1991959011
C12	-6.2966282189	1.2448897611	-0.0354676198	C12	-0.0241807389	-0.1617009127	0.2887947812
N13	-4.9393142401	1.4586368502	-0.0570261511	C13	-6.9417217801	-0.1455894860	-0.0814467436
N14	-2.0090515349	1.3393786601	0.0764363119	C14	1.4708314022	-0.1701347900	0.3644562968
C15	-0.6633155687	1.1205260568	0.0777208175	C15	-0.6570138159	-1.4146006781	0.2860582522
C16	0.0653305492	2.3784388559	-0.0469923815	C16	-6.2816044412	-1.3910179426	-0.0425130008
C17	-0.8670940623	3.3542880974	-0.1084896311	C17	-6.9902227194	-2.6672223020	-0.0738249044
C18	-0.0233753682	-0.1332576083	0.1667620239	C18	-6.0453533195	-3.6276179302	0.0138253385
C19	-6.9482540211	0.0033832193	-0.0722240292	C19	-4.7627885359	-2.9348441888	0.0928384390
C20	1.4701950631	-0.1605146477	0.2459173116	N20	-4.9366195601	-1.5826436569	0.0508709606
C21	-0.6771750617	-1.3746554114	0.1873193057	N21	-2.0074554283	-1.6511516433	0.1858549449
C22	-8.4457417373	0.0312987406	-0.0938322550	C22	-2.2694385954	-2.9962281630	0.2568798188

C23	-6.3049485573	-1.2509561514	-0.0873260178	C23	-1.0064943022	-3.6521948510	0.4090354846
C24	-7.0267918573	-2.5117740067	-0.2203238244	C24	-0.0301962957	-2.6951013982	0.4271846980
C25	-6.0941911667	-3.4893073444	-0.1923166662	C25	-3.5325140022	-3.6090651305	0.2044478267
C26	-4.8050179717	-2.8236517164	-0.0393208109	C26	2.1363064099	0.2763832393	1.5125307274
N27	-4.9617614142	-1.4695485096	0.0053142338	C27	3.5239952293	0.2629421576	1.5788503597
N28	-2.0295476570	-1.5891559971	0.0750024191	C28	4.2840931019	-0.1928064071	0.4960856854
C29	-2.3158312666	-2.9322532558	0.1279620287	C29	3.6256716041	-0.6410111704	-0.6596672115
C30	-1.0631705745	-3.6072413369	0.2935387084	C30	2.2410862023	-0.6305555403	-0.7167458460
C31	-0.0726861166	-2.6656254205	0.3323600354	N31	5.6835225790	-0.1582385043	0.6727213360
C32	-3.5847305295	-3.5238439477	0.0454988648	N32	6.3546933851	-0.5642967501	-0.3195305223
C33	-3.6257004380	-5.0213843986	0.0530585994	C33	7.7161847063	-0.5351830450	-0.1581174529
C34	2.1389790399	0.3447376553	1.3679523842	N34	8.4876788927	-0.9674636536	-1.2190159309
C35	3.5260949072	0.3174033859	1.4404494324	C35	9.8051361238	-0.8751696746	-0.8989018202
C36	4.2836145902	-0.2107040041	0.3890557351	C36	9.8963550937	-0.3742151871	0.3938653610
C37	3.6229940140	-0.7169344567	-0.7414932336	C37	8.5880256371	-0.1591253472	0.8633236525
C38	2.2389547945	-0.6922641279	-0.8039524866	C38	-3.5549784928	-5.0932688117	0.3079834345
C39	-3.1691318795	-5.7590033709	-1.0468401848	C39	-8.4277559878	-0.1412648191	-0.1580156771
C40	-3.2103828282	-7.1487509226	-1.0414867460	C40	-3.4335680281	4.7888456513	0.2197565567
C41	-3.7028256925	-7.8547848502	0.0619677364	S41	-3.3553510785	-6.1214627841	-1.0954668828
C42	-4.1543998132	-7.1170537173	1.1583638999	C42	-3.5054059847	-7.5559781115	-0.1368003134
C43	-4.1194963531	-5.7241602379	1.1559809186	C43	-3.6986009481	-7.2603498446	1.1805821038
C44	-9.1870535278	-0.3893575006	1.0181548694	C44	-3.7274438137	-5.8567570832	1.4308324607
C45	-10.5768457913	-0.3621078454	1.0008674325	S45	-9.2682757295	-0.1042472808	-1.6938445448
C46	-11.2795768373	0.0813610270	-0.1254049581	C46	-10.8138917914	-0.1235432452	-0.9132823239
C47	-10.5384265120	0.4986395901	-1.2328526230	C47	-10.6892152465	-0.1560175936	0.4445533023
C48	-9.1451913397	0.4774406752	-1.2190734880	C48	-9.3286884459	-0.1655038796	0.8719903254
C49	-3.2046971531	9.2315115202	-0.1183493377	S49	-3.3820146389	5.7745435242	-1.2266505205
N50	5.6823612199	-0.1832656738	0.5688648671	C50	-3.3825407052	7.2369857952	-0.2988863656
C51	-3.7420064182	-9.3638140015	0.0579426938	C51	-3.4200008645	6.9809158376	1.0401672733
C52	-12.7887917332	0.1040543773	-0.1338831544	C52	-3.4492604552	5.5854389316	1.3326028084
N53	6.3528713884	-0.6576485966	-0.3927745314	H53	-6.1145871372	4.4175733558	0.0576692511
C54	7.7144522060	-0.6357706372	-0.2239400302	H54	-8.0183347323	2.5373721286	-0.0927938067
N55	8.4865322340	-1.1422831473	-1.2506340762	H55	-4.2548358812	0.6188418127	0.0945045795
C56	9.8035955843	-1.0472105500	-0.9281766470	H56	1.0906070436	2.4922918716	0.2174941347
C57	9.8934125607	-0.4688662293	0.3318023210	H57	-0.7943351202	4.3887772038	0.1820386123
C58	8.5846873258	-0.2087093255	0.7783948708	H58	-8.0588576995	-2.7901307434	-0.1477922055
H59	-2.6106990208	5.0833274183	1.9551011468	H59	-6.1823953249	-4.6969973602	0.0239147240
H60	-2.5373076386	7.5428038331	1.9189572572	H60	-2.7128300650	-0.9294139295	0.0987384529
H61	-3.9843544604	7.5247927412	-2.1145750859	H61	-0.8818576364	-4.7179785665	0.5049733012
H62	-4.0604741554	5.0638117460	-2.0748904279	H62	1.0298070184	-2.8473872692	0.5437028595
H63	-6.0349332835	4.5497425944	0.0905252924	H63	1.5584365366	0.6276464178	2.3593686046
H64	-7.9727989150	2.7079040672	0.0763428863	H64	4.0471982930	0.6000848186	2.4656753076
H65	-4.2424994309	0.7234260982	-0.0490221498	H65	4.2156756917	-0.9867729355	-1.4977880886
H66	1.1365408890	2.4850021363	-0.1025004993	H66	1.7383499234	-0.9707370864	-1.6151899667
H67	-0.7079683102	4.4143873666	-0.2216076375	H67	8.0955185323	-1.2944917253	-2.0875057587
H68	-8.0932778059	-2.6176376089	-0.3382611086	H68	10.5762995782	-1.1646244677	-1.5940688016
H69	-6.2473926317	-4.5525856374	-0.2823388315	H69	10.8146617069	-0.1887042817	0.9286718978
H70	-2.7238537929	-0.8541574082	0.0134070688	H70	8.2792745372	0.2218450799	1.8222325929
H71	-0.9529554106	-4.6748558821	0.3855256472	H71	-3.4427242085	-8.5257455390	-0.6060634773
H72	0.9828783968	-2.8355965460	0.4632086212	H72	-3.8163129372	-8.0128736026	1.9491004298
H73	1.5625871265	0.7528929906	2.1898367511	H73	-3.8697915756	-5.4193763242	2.4104841313
H74	4.0510493135	0.6992701279	2.3078577701	H74	-11.7159119040	-0.1110998762	-1.5055717274
H75	4.2111300910	-1.1171121183	-1.5564485300	H75	-11.5341999471	-0.1733184546	1.1201382658
H76	1.7342610043	-1.0761068832	-1.6834411421	H76	-9.0201594625	-0.1921064349	1.9090139122
H77	-2.7896254566	-5.2345424893	-1.9165986779	H77	-3.3537981827	8.1921142720	-0.8003682477
H78	-2.8574588299	-7.6943199428	-1.9112905752	H78	-3.4257904561	7.7559550691	1.7951580622
H79	-4.5378297645	-7.6369970031	2.0303451891	H79	-3.4778311826	5.1776159653	2.3346679318
H80	-4.4732772920	-5.1749730716	2.0211316650				
H81	-8.6649787228	-0.7341420399	1.9036284962				
H82	-11.1254384039	-0.6889325210	1.8789001047				
H83	-11.0555782237	0.8422289958	-2.1229275321				
H84	-8.5934134045	0.7997381952	-2.0950765461				
H85	-3.5355134605	9.6306597820	-1.0791096481				
H86	-3.8448292006	9.6579530388	0.6603223052				
H87	-2.1886424401	9.5952537844	0.0637316764				
H88	-4.1495262370	-9.7537067366	0.9926594184				
H89	-4.3618123559	-9.7415612127	-0.7612546370				
H90	-2.7408607548	-9.7861348903	-0.0732458890				
H91	-13.1758880724	0.4730245673	-1.0856265890				
H92	-13.2010560093	-0.8963618864	0.0304760396				
H93	-13.1796024982	0.7487059872	0.6595962151				
H94	8.0949038026	-1.5172294581	-2.0997691510				
H95	10.5747231861	-1.3887367776	-1.5994226249				
H96	10.8107840499	-0.2623196919	0.8604666114				
H97	8.2748708818	0.2342062486	1.7099028921				

6

E = -2226.0471

Nimag = 0

C1	-3.3282143794	3.3865842264	0.0400664325
C2	-4.6110719694	2.8110074341	0.0619006371
C3	-2.1134326300	2.6646974197	-0.0095967982
C4	-5.8657062361	3.4916037742	-0.0394461614
C5	-6.8631133733	2.5593928721	0.0257041274
C6	-6.2604533828	1.2681680342	0.1611596027
N7	-4.9023836629	1.4718116578	0.1708145392
N8	-1.9956047269	1.3154407104	-0.1353254402
C9	-0.6533474047	1.0636638197	-0.1363433254
C10	0.1057135936	2.2997763709	0.0133356228
C11	-0.8021276218	3.2960505786	0.0926652724
C12	-0.0430779526	-0.1993013801	-0.2431370797
C13	-6.9274634206	0.0299755654	0.2204058243
C14	1.4550114918	-0.2533014833	-0.2574313687
C15	-0.7145707329	-1.4277031921	-0.3206175048
C16	-6.3134401187	-1.2382878580	0.1344711279
C17	-7.0617371388	-2.4910152992	0.1670119617
C18	-6.1585734221	-3.4775859821	-0.0058906755
C19	-4.8558575436	-2.8289180672	-0.1095027382
N20	-4.9802331547	-1.4740987059	-0.0181430790
N21	-2.0758031876	-1.6270046733	-0.3070278303
C22	-2.3684440093	-2.9664526244	-0.3509227723
C23	-1.1150380109	-3.6568581039	-0.4058922333
C24	-0.1148582110	-2.7246566130	-0.3960652139
C25	-3.6519469271	-3.5407843076	-0.2914986834
C26	2.1747775639	0.1247723015	-1.3963412052
C27	3.5641673903	0.0868845604	-1.4011614211
C28	4.2682497681	-0.3240262273	-0.2639796954
C29	3.5536091942	-0.7074712445	0.8817806297
C30	2.1681118536	-0.6731196271	0.8774424800
N31	5.6752143727	-0.3114526521	-0.3773506452
N32	6.2956619380	-0.6550769503	0.6694921299
C33	7.6636328615	-0.6423252945	0.5710476518
C34	-3.7131882761	-5.0048939121	-0.4426507496
C35	-8.3882318303	0.1008949062	0.3866653564
C36	-3.2731282831	4.8536210738	0.0970809976
O37	-4.3104120314	-5.7379135134	0.5527726080
C38	-4.2536267034	-7.0470188620	0.1843694622
C39	-3.6376646787	-7.1757688951	-1.0188629740
C40	-3.2862141356	-5.8506694329	-1.4267245283
O41	-9.1851041361	-0.5666855688	-0.5103551667
C42	-10.4778300897	-0.3566942726	-0.1421549079
C43	-10.5358143954	0.4262592937	0.9661209289
C44	-9.1806254874	0.7244283861	1.3098361569
O45	-2.5126750081	5.5095533826	-0.8397027155
C46	-2.6059975484	6.8412710626	-0.5812165264
C47	-3.4026351717	7.0623427738	0.4966838860
C48	-3.8359343557	5.7740515062	0.9379243528
N49	8.3809664985	-1.0026575216	1.6947484453
C50	9.7129186978	-0.9356213894	1.4335550536
C51	9.8689482864	-0.5249709755	0.1154428991
C52	8.5855223891	-0.3393190098	-0.4304067841
H53	-5.9748909164	4.5530461158	-0.1814838305
H54	-7.9226940842	2.7324808010	-0.0530924433
H55	-4.2066420624	0.7359743812	0.2073478644
H56	1.1798346513	2.3793597998	0.0611877032
H57	-0.6143023156	4.3494225075	0.2148822732
H58	-8.1271671597	-2.5898794821	0.2858678562
H59	-6.3428584359	-4.5376322638	-0.0457896583
H60	-2.7700042500	-0.8890225938	-0.2769027088
H61	-1.0084944504	-4.7277970887	-0.4271346198
H62	0.9466831324	-2.9066019793	-0.4216385884
H63	1.6392865151	0.4493426512	-2.2811156738
H64	4.1312501071	0.3751710478	-2.2782541369
H65	4.1012688535	-1.0208899668	1.7603418208
H66	1.6199650040	-0.9621306492	1.7673477438
H67	-4.6852876380	-7.7480064727	0.8783707622
H68	-3.4601700022	-8.0947983804	-1.5542338621
H69	-2.8019759581	-5.5554835277	-2.3434736952
H70	-11.2234105880	-0.8222755858	-0.7639815360
H71	-11.4280955406	0.7470433655	1.4796164030

7

E = -2504.3704

Nimag = 0

C1	-2.8365789704	5.5664160478	-1.0246750239
C2	-3.2212680609	4.8271667711	0.1013700477
C3	-2.7569096212	6.9534198175	-0.9745661633
C4	-3.0536213155	7.6549097426	0.1996839909
C5	-3.4357076018	6.9155755212	1.3209427928
C6	-3.5212163845	5.5255120529	1.2745776386
C7	-3.3071969082	3.3325158321	0.0472790264
C8	-4.5938844797	2.7762630712	0.0936996040
C9	-2.1066932903	2.6002640861	-0.0456969956
C10	-5.8374147828	3.4871862446	0.1309875566
C11	-6.8513372763	2.5712932681	0.1789386423
C12	-6.2709670556	1.2613577054	0.1726857165
N13	-4.9097812539	1.4392753492	0.1126666434
N14	-1.9937140431	1.2472136245	-0.1777265574
C15	-0.6554061550	0.9935079654	-0.2420455062
C16	0.1117575821	2.2287829642	-0.1207817498
C17	-0.7904844034	3.2272006920	0.0000423136
C18	-0.0548927953	-0.2742081875	-0.3891330649
C19	-6.9531823967	0.0369171122	0.2166855768
C20	1.4340415632	-0.3430274377	-0.5179417824
C21	-0.7447733012	-1.4959346754	-0.4219789289
C22	-8.4443431155	0.1041383089	0.3428064508
C23	-6.3463218497	-1.2333861490	0.1497180470
C24	-7.0943071947	-2.4792190189	0.2792711749
C25	-6.1933973113	-3.4787743724	0.1568578874
C26	-4.8969921967	-2.8412957790	-0.0467505632
N27	-5.0179768217	-1.4830336574	-0.0321564180
N28	-2.0971504132	-1.6771027437	-0.2611741347
C29	-2.4236401644	-3.0092903589	-0.3468250318
C30	-1.1991280874	-3.7118253086	-0.5902031288
C31	-0.1841144255	-2.7970828108	-0.6365681463
C32	-3.7033826760	-3.5693563027	-0.2236481777
C33	-3.7882367039	-5.0638763886	-0.2867503222
C34	2.0851603711	0.1744757361	-1.6445714280
C35	3.4686296012	0.1065407608	-1.7586181382
C36	4.2381414150	-0.4781438514	-0.7476184314
C37	3.5960173420	-0.9964003536	0.3873143121
C38	2.2160434435	-0.9293268305	0.4924104502
C39	-3.3038562659	-5.8572881107	0.7611689437
C40	-3.3861279519	-7.2440618095	0.7035978239
C41	-3.9490702281	-7.8912104987	-0.4022450686
C42	-4.4283933365	-7.0978520159	-1.4467485774
C43	-4.3523943121	-5.7076718759	-1.3918172432
C44	-9.2734530676	-0.2545300883	-0.7279203322
C45	-10.6573091605	-0.1912865097	-0.6110576007
C46	-11.2663678244	0.2278792755	0.5773326786
C47	-10.4377160939	0.5835740462	1.6434562626
C48	-9.0500813175	0.5260488026	1.5299172761
C49	-2.9600509497	9.1608712154	0.2437267309
N50	5.6350856703	-0.4910509031	-0.9646572210
C51	-4.0316655098	-9.3975284902	-0.4554715875
C52	-12.7701122959	0.2895853719	0.6940319684
N53	6.3093779360	-1.0433305047	-0.0517996235
C54	7.6797285392	-1.0554624437	-0.2544302143
C55	8.6128752288	-1.6520265573	0.6718079336
C56	9.9044489112	-1.4650165612	0.1241097779
N57	9.7463832079	-0.7863792938	-1.0753434025
C58	8.4225031960	-0.5402838339	-1.3049299288
C59	8.4818490398	-2.3194185735	1.8963671496
C60	9.6268638275	-2.7752466651	2.5319800638
C61	10.9024654895	-2.5787122602	1.9700147863
C62	11.0602155596	-1.9210133108	0.7583320608
H63	-2.6038554983	5.0455776000	-1.9465669103
H64	-2.4607846756	7.5010116011	-1.8640606782
H65	-3.6664648567	7.4312859288	2.2475138960
H66	-3.8129474290	4.9745108621	2.1616142766
H67	-5.9269569030	4.5604870694	0.1117355482
H68	-7.9099061745	2.7692228527	0.2054117635
H69	-4.2353206704	0.6864541999	0.0465551833
H70	1.1868647363	2.3058833360	-0.1091133342
H71	-0.5982954103	4.2802019264	0.1280227028

H72	-8.8311497356	1.3046367719	2.1483968500
H73	-2.0591138190	7.4872300127	-1.2469221612
H74	-3.6468092427	8.0195929100	0.9287944596
H75	-4.4611299608	5.5504364530	1.7869354006
H76	7.9454743991	-1.2669990333	2.5639542489
H77	10.4477265450	-1.1780859112	2.1838366941
H78	10.8129654344	-0.3788198714	-0.3857161164
H79	8.3253276196	-0.0241211736	-1.4268488886

H72	-8.1545157844	-2.5615640663	0.4564677199
H73	-6.3708750556	-4.5405740575	0.2140584342
H74	-2.7660994783	-0.9262165199	-0.1401790873
H75	-1.1237278771	-4.7780998793	-0.7237759264
H76	0.8601203901	-2.9912071509	-0.8161716889
H77	1.4984569098	0.6248616075	-2.4366527762
H78	3.9797002567	0.4984444195	-2.6300595410
H79	4.1964226328	-1.4386359690	1.1708601022
H80	1.7255001412	-1.3226448598	1.3757523848
H81	-2.8692140642	-5.3788717663	1.6316985184
H82	-3.0096369581	-7.8337295750	1.5338316036
H83	-4.8665518265	-7.5715650803	-2.3192844522
H84	-4.7295213111	-5.1144958556	-2.2171744203
H85	-8.8250155578	-0.5805637119	-1.6595294186
H86	-11.2753754629	-0.4704111758	-1.4589057966
H87	-10.8810918286	0.9069491231	2.5797131392
H88	-8.4291782953	0.8003507861	2.3753783944
H89	-3.2241060914	9.5472670488	1.2300348049
H90	-1.9464902007	9.5017875895	0.0106295012
H91	-3.6318199010	9.6210674579	-0.4876207625
H92	-4.4969324293	-9.7379642280	-1.3825327489
H93	-3.0377314936	-9.8514993935	-0.3928870259
H94	-4.6198519953	-9.7915236894	0.3792118782
H95	-13.0786419088	0.6331847483	1.6832605695
H96	-13.1971979532	0.9730060847	-0.0465595924
H97	-13.2213207036	-0.6930704952	0.5247708804
H98	10.4960782700	-0.5120235432	-1.6888593134
H99	8.0803235195	-0.0214291609	-2.1842299286
H100	7.5013267092	-2.4716450543	2.3301632782
H101	9.5425398487	-3.2934309462	3.4802635976
H102	11.7771524597	-2.9476448013	2.4928760752
H103	12.0425377515	-1.7695167601	0.3247892520

8

E = -3348.6749

Nimag = 0

C1	-3.2823286349	3.2304226128	-0.0874735166
C2	-4.5722363110	2.6858567117	-0.1357307057
C3	-2.0846471996	2.4919014966	0.0263956946
C4	-5.8072205522	3.4073212444	-0.2324067159
C5	-6.8290991304	2.5010418372	-0.2477229427
C6	-6.2613875490	1.1876988619	-0.1606632836
N7	-4.9001569226	1.3522863223	-0.0966981174
N8	-1.9833075801	1.1393766688	0.1473310878
C9	-0.6470905292	0.8797355037	0.2494426514
C10	0.1273414858	2.1142488823	0.1703170377
C11	-0.7674498554	3.1170593894	0.0349945647
C12	-0.0565594051	-0.3907335250	0.4017229458
C13	-6.9570643081	-0.0295270745	-0.1377823153
C14	1.4287207167	-0.4603377280	0.5801301484
C15	-0.7526474654	-1.6088313350	0.4243176200
C16	-6.3642872289	-1.3046250649	-0.0364771941
C17	-7.1392368100	-2.5416285506	-0.0130788648
C18	-6.2462472561	-3.5461279106	0.1138903995
C19	-4.9288799528	-2.9192632915	0.1686667662
N20	-5.0312187933	-1.5629230312	0.0684154696
N21	-2.1093943939	-1.7788864088	0.2801502705
C22	-2.4449265366	-3.1049623588	0.3884749287
C23	-1.2236703241	-3.8200030576	0.6054155761
C24	-0.1997969716	-2.9141620320	0.6314854900
C25	-3.7373780754	-3.6523994755	0.3246896706
C26	2.0237447913	0.0069360910	1.7580096291
C27	3.4014368038	-0.0490824554	1.9274107367
C28	4.2216373282	-0.5673251486	0.9203247027
C29	3.6349083169	-1.0403060711	-0.2632633854
C30	2.2584365554	-0.9882246471	-0.4230331910
N31	5.6077775372	-0.5644466716	1.1977242734
N32	6.3381012634	-1.0038520054	0.2667557037
C33	7.6973014962	-0.9992871873	0.5329599482
C34	8.6934587852	-1.4485413449	-0.4105369443
C35	9.9504396848	-1.2888600273	0.2196324367
N36	9.7123959093	-0.7678421240	1.4832806517
C37	8.3716359819	-0.5933129104	1.6740612315
C38	-3.8399419128	-5.1291427071	0.4784427938
C39	-8.4417591559	0.0520595634	-0.1948980275

C40	-3.1816082180	4.7144292003	-0.1331567365	
S41	-3.7039186257	-6.2125846698	-0.8903189775	
C42	-3.9271698741	-7.6041639433	0.1162137885	
C43	-4.0966083079	-7.2549517747	1.4238181416	
C44	-4.0470267118	-5.8443552042	1.6270780387	
S45	-9.3093102255	0.0531473887	-1.7160023221	
C46	-10.8382096784	0.1608338214	-0.9097970421	
C47	-10.6887572992	0.1939527303	0.4455783603	
C48	-9.3225667383	0.1322842186	0.8497798652	
S49	-3.1321554670	5.5929824923	-1.6471039791	
C50	-3.0056714068	7.1128995726	-0.8264527454	
C51	-2.9999080942	6.9522909911	0.5280356728	
C52	-3.0994144214	5.5848965086	0.9200328261	
C53	8.6442915909	-1.9625383199	-1.7124757168	
C54	9.8337517096	-2.2986696567	-2.3407757735	
C55	11.0736847508	-2.1321264054	-1.6960003341	
C56	11.1504748496	-1.6249393315	-0.4067587868	
H57	-5.8813765873	4.4811345830	-0.2791961108	
H58	-7.8854842860	2.7032427579	-0.3088459829	
H59	-4.2330861420	0.5952749335	-0.0068745850	
H60	1.2022913428	2.1879278138	0.2014373344	
H61	-0.5721446137	4.1732480661	-0.0581895988	
H62	-8.2129498129	-2.6110315515	-0.0818311702	
H63	-6.4397774176	-4.6053949885	0.1686571315	
H64	-2.7717514448	-1.0239142047	0.1479383912	
H65	-1.1589760558	-4.8873470408	0.7373839353	
H66	0.8461942768	-3.1171860305	0.7899100944	
H67	1.3981841259	0.4112713137	2.5451828180	
H68	3.8698459605	0.3061176211	2.8376519131	
H69	4.2737128307	-1.4357513357	-1.0416111970	
H70	1.8116910419	-1.3466015716	-1.3437052511	
H71	10.4223456392	-0.5493814644	2.1630138472	
H72	7.9698451578	-0.1976109701	2.5913841459	
H73	-3.9205128998	-8.5909068602	-0.3208762235	
H74	-4.2515700641	-7.9741209746	2.2172743108	
H75	-4.1601818773	-5.3673310158	2.5919621997	
H76	-11.7496468319	0.1918904801	-1.4867585360	
H77	-11.5198541793	0.2595153518	1.1353534487	
H78	-8.9955165183	0.1446627288	1.8813678087	
H79	-2.9427719172	8.0277909378	-1.3953672625	
H80	-2.9276500076	7.7763890997	1.2254582605	
H81	-3.1090711565	5.2493824764	1.9489179453	
H82	7.6914677963	-2.0908515962	-2.2109365189	
H83	9.8127529960	-2.6974102782	-3.3484587523	
H84	11.9848815173	-2.4045453296	-2.2157347696	
H85	12.1054957008	-1.4961072090	0.0904146554	
